# Informing the Development of Telehealth Education in Physiotherapy Programs. Assessments and Interventions for Individuals Accessing Physiotherapy Care via Synchronous Telehealth. A Scoping Review

**DOI:** 10.1002/msc.70039

**Published:** 2025-01-09

**Authors:** Luke M. Davies, Vidya Lawton, Rebecca Bevan, Mikayla Mestousis, Verity Pacey

**Affiliations:** ^1^ Department of Health Sciences Faculty of Medicine, Health and Human Sciences Macquarie University Sydney Australia; ^2^ School of Primary and Allied Health Care Monash University Peninsula Victoria Australia

**Keywords:** assessment, physical therapy, physiotherapy, synchronous, telehealth, treatment, videoconference

## Abstract

**Background:**

In order to develop contemporary telehealth curricula for entry‐to‐practice physiotherapy programs that develop the capabilities required to practice telehealth, it is important to evaluate the delivery of telehealth practices within the physiotherapy profession.

**Objective:**

To assess the current literature to (i) determine what types of assessments and interventions have been delivered via synchronous forms of telehealth (videoconferencing and telephone) by physiotherapists (ii) determine which platforms were used for service delivery and which practice areas have delivered synchronous telehealth physiotherapy assessments and interventions.

**Design:**

Scoping review adhering to Joanna Briggs Institute guidelines. Three electronic databases (Medline, Embase, and CINAHL) were searched for articles involving physiotherapists using synchronous forms of telehealth (videoconferencing/telephone) to deliver assessments and/or interventions.

**Study Selection:**

Articles were included if they related to physiotherapists using synchronous forms of telehealth (videoconferencing and/or telephone) to deliver assessments and/or interventions.

**Data Synthesis:**

A narrative synthesis was conducted.

**Results:**

From 2748 records, 134 studies were included. Standardised assessments (e.g. subjective assessments, range of motion, functional assessment) were the most commonly (82%) delivered. Most interventions delivered were exercise (82%), followed by education (27%). Freely available synchronous videoconferencing platforms including Zoom (*n* = 30, 34%), WhatsApp (*n* = 10, 11%), and Skype (*n* = 9, 10%) were commonly used. Telehealth‐delivered physiotherapy assessment and interventions were primarily utilised in musculoskeletal (33%), neurological (23%) and respiratory (14%) practice areas.

**Conclusion:**

The diverse use of physiotherapy telehealth‐delivered assessments and interventions across varied clinical practice settings and patient populations highlights the importance of preparing future physiotherapists with telehealth skills fit for contemporary practice.

## 1 Introduction

Historically, physiotherapy consultations occur in‐person involving a ‘hands on’ approach to patient assessment and treatment (Moffatt and Kerry 2018). However, physiotherapy services delivered via telehealth, defined by the World Health Organization as ‘the use of telecommunications and virtual technology to deliver healthcare outside of traditional healthcare facilities’ (Telehealth Services Australia 2020), appear to be increasing globally. This was particularly evident during and after the COVID‐19 pandemic, when telehealth practices were widely adopted to maintain clinical services (Haines and Berney 2020; MacDonald et al. 2020).

Telehealth is now considered an important aspect of contemporary physiotherapy practice (M. Cottrell et al. 2021), and research exploring the benefits and utility of telehealth delivered physiotherapy management has been growing (Hawley‐Hague et al. 2023; Suso‐Marti et al. 2021). In particular, research has focused on the feasibility for physiotherapists to perform certain clinical assessments using synchronous forms of telehealth such as videoconference (Zischke et al. 2021). Additionally, when compared to in‐person care, telehealth delivered physiotherapy has been shown to provide similar, and in some instances greater improvements in clinical outcomes such as pain, disability, functional outcomes, as well as quality of life for a variety of populations including surgical patients (van Egmond et al. 2018), individuals with musculoskeletal (M. A. Cottrell et al. 2017), neurological disorders (Seron et al. 2021), and respiratory disorders (Tsai et al. 2017).

Despite the growing body of evidence demonstrating the benefits and utility of telehealth delivered physiotherapy management, the physiotherapy pre‐registration curriculum has typically focused on managing patients in‐person. There is an absence of telehealth education and training observed within physiotherapy programs (Davies, Hinman, et al. 2023; Davies, Lawford, et al., 2023; Grant, Jones, and Land 2022) despite consistent evidence that telehealth can provide comparable patient outcomes (Hawley‐Hague et al. 2023). A lack of telehealth guidelines and standards specific to physiotherapy on a global scale (Hui et al. 2021) coupled with a lack of confidence and expertise amongst educators responsible for developing telehealth education (Davies, Lawford, et al. 2023; Govender and Mars 2018) may be contributing to the absence of telehealth within the physiotherapy curriculum.

In order to develop contemporary telehealth curricula that develop the capabilities required to practice telehealth, it is important to evaluate the delivery of telehealth practices within the physiotherapy profession. To our knowledge, prior reviews investigating telehealth‐delivered physiotherapy assessments have only considered literature published up to 2020 (Zischke et al. 2021) prior to the COVID‐19 pandemic. More recently, a review published investigating digital physiotherapy assessment versus conventional in‐person assessment only considered literature published up until 2022 however only focused on one clinical population (musculoskeletal disorders) (Bernhardsson et al. 2023). Additionally, reviews exploring telehealth‐delivered physiotherapy interventions focus on specific populations and interventions, such as the delivery of exercises (Dias et al. 2021; Gava et al. 2022; Hirohama et al. 2023), often failing to capture not only the breadth of interventions delivered by physiotherapists, but also the breadth of patient populations accessing telehealth care. Given the rapid expansion of telehealth practice and research, it is important to have a comprehensive understanding of telehealth use in physiotherapy practice to facilitate the development of contemporary telehealth curricula.

Thus, the aim of this scoping review is to inform telehealth education and training in entry‐to‐practice physiotherapy programs by determining the types of synchronously delivered assessments and interventions that are performed by physiotherapists. Secondary aims were to determine which platforms were used for service delivery and which clinical practice areas have utilised synchronous telehealth delivered assessments and interventions by physiotherapists.

## 2 Methods

This scoping review was conducted in accordance with the JBI methodology for scoping reviews (Peters et al. 2017). We reported the review in line with the Preferred reporting Items for Systematic Reviews and Meta‐Analyses reporting guidelines for scoping reviews (PRISMAS‐ScR) (Tricco et al. 2018). A protocol for this scoping review was registered on the Open Sciences Framework on 24th of June 2024 https://doi.org/10.17605/OSF.IO/QZ4YU.

## 3 Eligibility Criteria

Studies were eligible for inclusion if they were published in English and involved physiotherapists using synchronous forms of telehealth (videoconferencing and/or telephone) to deliver assessments and/or interventions. Validity studies, reliability studies, quasi‐experimental study designs including randomised controlled trials, non‐randomised controlled trials, qualitative studies where sufficient detail regarding the interventions are available, case studies/series, crossover designs, and observational study designs were considered for inclusion.

Studies were excluded if the assessment and/or intervention used only asynchronous forms of telehealth or virtual reality technology, had insufficient detail on the mode of telehealth used, or were not delivered by a physiotherapist. Commentary papers, systematic/scoping/narrative reviews, study protocols, poster presentations, abstracts, opinion pieces and/or editorial papers were also excluded.

## 4 Information Sources and Search Strategy

An initial limited search of PubMed and Google Scholar was conducted on 25^th^ March 2024 to identify articles on the topics of ‘Physiotherapy assessment’ and ‘Physiotherapy interventions’ delivered via synchronous forms of telehealth. Following the initial search, a comprehensive search strategy was developed in conjunction with a research librarian with extensive expertise in the development of search strategies for scoping reviews. Three databases were searched, including Medline, Embase, and CINHAL. The full electronic search strategy for Medline is presented in Table 1, which was subsequently adapted for each of the included databases. To maintain currency, the search was conducted from 2013 up to the 1st of April 2024. Studies meeting the eligibility criteria were uploaded into Endnote (EndNote 20, Clarivate, Philadelphia, PA) and then transferred to Covidence (Veritas Health Innovations, Melbourne, Australia) for de‐duplication and screening.

**TABLE 1:** Full Medline search strategy.

Search	Query
1	Physical therapists/
2	(physical therap* or physiotherap*).ti,ab.
3	exp physical therapy modalities/
4	or/1–3
5	Telemedicine/or telerehabilitation/or remote consultation/or (telehealth or teleconsulation or tele‐consultation or teletreatment or tele‐treatment or telecare or tele‐care)
6	And/4–5
7	Limit 6 to (English language and yr = ‘2013‐Current’)

## 5 Selection of Sources of Evidence

Two reviewers (L.M.D. and V.P.) independently screened the titles and abstracts of papers based on eligibility criteria. Any disagreements were resolved by robust discussion between the two reviewers to reach a consensus. Full texts were independently screened by two of five reviewers (L.M.D., V.P., V.L., R.B. and M.M.). Any concerns raised regarding the eligibility of studies were resolved among the research team to reach consensus. Considerable efforts were made to retrieve full‐text records through various physical and digital sources, including two university libraries. Due to the broad nature of the scoping review, and the volume of studies, we did not use citation chaining methods.

## 6 Data Charting

Data from eligible studies were extracted into a purpose‐built spreadsheet in Microsoft Excel (Microsoft Corporation, United States of America). Data items included first author, year of publication, country of origin (where participants were located), study design, participant details and sample size, healthcare service/providers, participant health condition(s), assessment(s), intervention(s), mode(s) of delivery (hybrid or synchronous only), type of synchronous telehealth (videoconference/telephone) and, where relevant, platform(s) used. Data were extracted independently by all members of the research team. Following data extraction, the research team independently reviewed 10% of another team member data sets, resulting in cross‐checking of 50% of entries. This was performed to ensure consistency and credibility of the extracted data.

## 7 Data Analysis

Data were summarised descriptively using frequencies and percentages to categorise the published literature (e.g., country, population, type of study, and the mode of synchronous telehealth). Additionally, content analysis was performed by two research team members (L.M.D. and V.P.) and used to provide a narrative summary on the constructs of assessments, interventions, and clinical practice areas to address the review aims.

## 8 Results

**FIGURE 1:**
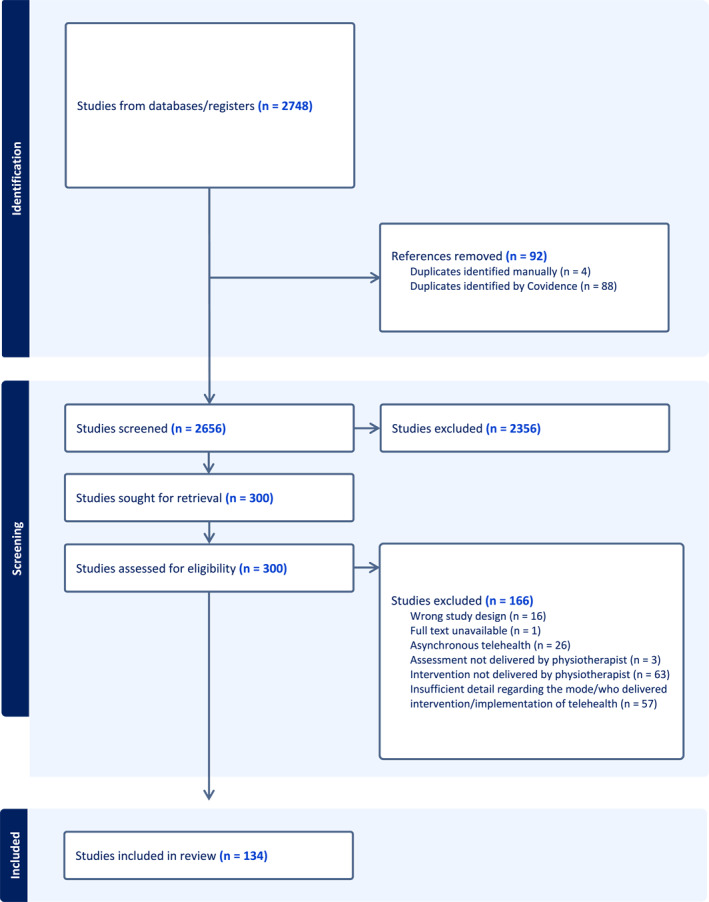
PRISMA flow diagram.

A total of 2748 records were identified through the database searches. After duplicates were removed and title and abstracts were screened, 300 papers were downloaded for full text screening, with a final 134 studies included in the review (Figure 1). The most common reasons for exclusion were that the intervention was not delivered by a physiotherapist and insufficient detail regarding the mode of delivery and/or who delivered the intervention. Table 2 describes the characteristics of the included studies.

**TABLE 2:** Study characteristics.

	*n*, (%)
Country, (*n* = 136)^a^	
Australia	27 (20)
Canada	9 (7)
Spain	7 (5)
Turkey	19 (14)
United States of America	26 (19)
Other^b^	48 (35)
Study type, (*n* = 134)	
Randomised controlled trial	46 (34)
Prospective cohort	34 (25)
Cross‐sectional (reliability)	14 (10)
Retrospective cohort	3 (2)
Case series/studies	12 (9)
Feasibility/pilot study	23 (17)
Qualitative	2 (1)
Publication year, (*n* = 134)	
2013–2016	12 (10)
2017–2020	26 (19)
2021–2024	96 (71)
Clinical practice area, (*n* = 134)	
Musculoskeletal	43 (32)
Neurological	31 (23)
Respiratory	19 (15)
Oncology	9 (7)
Pelvic health	8 (6)
Healthy population	8 (6)
Cardiac	6 (4)
Metabolic	6 (4)
Other	4 (3)
Age of participants, (*n* = 134)^c^	
0–20 years	13 (9)
21–40 years	25 (19)
41–60 years	47 (35)
61+ years	51 (73)
Telehealth equipment, (*n* = 134)^c^	
Videoconference	106 (79)
Telephone	30 (22)
Web	2 (1)
Not reported	3 (2)
Mode of service delivery, (*n* = 134)	
Synchronous	83 (62)
Face‐to‐face and synchronous	24 (18)
Synchronous and asynchronous	18 (13)
Face‐to‐face, synchronous and asynchronous	9 (7)

^a^ Top 5 countries.

^b^ Belgium (3), Brazil (4), Czech Republic (2), Denmark (2), France (1), Greece (2), India (2), Ireland (3), Italy (3), Japan (2), Korea (2), Malawi (1), Malaysia (1), Nepal (1), Netherlands (3), Norway (4), not reported (6), Romania (1), Saudi Arabia (2), Sweden (1), United Kingdom (2).

^c^ Will not add to 100% as multiple age groups and technologies used across studies.

Of the 134 studies included in this review (Supporting Information S1), the majority were randomised controlled trials (*n* = 46, 34%) followed by prospective cohort studies (*n* = 34, 25%), with most articles published between 2021 and 2024 (*n* = 96, 71%). Most telehealth delivered physiotherapy assessments and interventions were administered to individuals over 41 years of age (*n* = 98, 72%). Of those assessments and/or interventions delivered via videoconference (*n* = 87), freely available platforms such as Zoom (*n* = 30, 34%), WhatsApp (*n* = 10, 11%), and Skype (*n* = 9, 10%) were commonly used (Supporting Information S2). Synchronous only delivery was the most common method of delivery (*n* = 84, 63%) rather than using a hybrid approach (*n* = 50, 37%). Videoconference was the preferred method of delivery for assessments (*n* = 42, 89%) and interventions (*n* = 65, 75%).

### 8.1 Assessments

Of the 28 assessments performed within 48 studies (Supporting Information S3), the most common type of assessment performed via telehealth was standardised assessments (*n* = 23, 82%). These included subjective assessments, range of motion, standardised postural examination, muscle length tests, straight leg raise, timed up and go, 30 s sit to stand, 5 m walk test, as well as the 2‐min step test, and were utilised across multiple practice areas. Orthopaedic tests such as self‐administered manual muscle and modified manual special tests were less frequently used (*n* = 5, 18%).

### 8.2 Interventions

Exercise based interventions were the most commonly delivered (*n* = 110, 82%) followed by education (*n* = 37, 27%) and coaching (*n* = 29, 21%). Strength‐ and aerobic‐based training were frequently prescribed across most studies. Additionally, range of motion, proprioception, deep breathing, postural control, flexibility, body weight exercises, and task‐orientated training were provided. Education regarding a patient's condition, performance of exercises, lifestyle modifications, the utilisation of feedback devices, as well as developing personal self‐management goals were frequently provided. Coaching typically involved counselling, self‐management support, encouragement, feedback on exercise programs and reinforcement on the value of lifestyle modification. A number of studies (*n* = 60, 45%) included in this review used a combination of exercise, education, and coaching (Supporting Information S4).

### 8.3 Practice Area

The most common clinical practice areas where physiotherapists delivered assessments and/or interventions were musculoskeletal (*n* = 43, 33%), neurological (*n* = 31, 23%), and respiratory (*n* = 19, 14%). Additional clinical practice areas receiving synchronous delivered assessments and/or interventions included oncology (*n* = 9, 7%), pelvic health (*n* = 8, 6%), healthy population (*n* = 8, 6%), cardiac (*n* = 6, 4%), metabolic (*n* = 6, 4%), and other (*n* = 4, 3%) areas, which included bleeding disorders, human immunodeficiency virus and burns (Supporting Information S5).

## 9 Discussion

Our findings demonstrate the breadth of physiotherapy practice and patient populations accessing telehealth delivered physiotherapy assessments and/or interventions. Across the globe, physiotherapists utilise freely available telehealth platforms across a wide range of clinical practice areas, patient conditions and age groups. The most common clinical practice areas were musculoskeletal, followed by neurological. Synchronously delivered standardised assessments and exercise, education and coaching were most commonly provided via telehealth. Given the growing use of telehealth by physiotherapists in multiple areas of practice, purposefully considered pre‐registration training in the use of telehealth will equip future graduates with work‐ready skills upon entering the profession.

Prior research shows that while many physiotherapy students and recent graduates believe that receiving pre‐professional telehealth training is beneficial (Martin, Mandrusiak, Russell, and Forbes 2022), they do not receive formal telehealth education, and lack confidence in providing consultations via telehealth (Davies, Hinman, et al. 2023; Davies, Lawford, and Chan 2024; Martin, Mandrusiak, Lang, et al. 2022). Interestingly, if provided with the opportunity, students and new graduates have reported interest in developing their telehealth delivery capabilities (Davies, Hinman, et al. 2023). Despite peak physiotherapy bodies advocating for the integration of telehealth into education and clinical practice (Michell et al. 2022; Shaw 2009; World Confederation for Physiotherapy and International Network of Physiotherapy Regulatory Authorities 2020), many entry‐to‐practice physiotherapy programs do not integrate formal telehealth education (Davies, Lawford, et al. 2023; Hui et al. 2021; Martin, Mandrusiak, Russell, and Forbes 2022). With the increased uptake of telehealth delivered physiotherapy services during the COVID‐ 19 pandemic, and the continuation of these services in clinical practice post pandemic (Albahrouh and Buabbas 2021; Bennell et al. 2021; Ezzat et al. 2023; Rausch et al. 2021; Reynolds, Awan, and Gallagher 2021), it is crucial to equip emerging and current physiotherapy workforces with the necessary capabilities to work in a telehealth environment.

Recently, updates to the Physiotherapy Practice Threshold for Australia and New Zealand and World Physiotherapy's Physiotherapist Education Framework have included digital practice and competence (Physiotherapy Board of Australia 2023; World Physiotherapy 2021), highlighting the importance placed on these capabilities within contemporary physiotherapy practice. The findings of this study have shown that although telehealth‐delivered physiotherapy assessments have been predominantly employed within the musculoskeletal domain, they have also been effectively applied across a range of clinical areas, including neurological, respiratory, cardiac, oncology, metabolic, and pelvic health conditions. Similarly, telehealth interventions have been delivered across the breadth of physiotherapy practice areas and age groups, particularly to older adults above the age of 60. Given the diverse practice areas and patient populations utilising telehealth‐delivered physiotherapy, these findings suggest that telehealth education should be purposefully scaffolded across all stages of entry‐level physiotherapy curricula.

The implications of our findings are relevant for physiotherapy educators who are responsible for developing and designing curricula in entry‐to‐practice programmes, as well as those responsible for enhancing the skills of practicing physiotherapists through ongoing education courses. Physiotherapy assessments and interventions identified above that are commonly used in practice across a range of different clinical practice areas should also be taught using telehealth delivery within physiotherapy programme curricula. Authentic assessments should be implemented at different stages of physiotherapy programs to enable reinforcement of foundational telehealth capabilities and the ability to adapt to perform assessments and interventions in a range of clinical areas. Case‐based scenarios that develop procedural skills through assessment of their application are a proven educational tool used in the classroom that can be adapted to telehealth assessments or interventions (Chéron et al. 2016). Case based scenarios using commonly identified free software such as ‘Zoom’ can mirror real practice as authentic high‐fidelity simulations have been shown to have superior educational outcomes (Eva et al. 2016).

Development of telehealth capabilities in physiotherapy students should be the shared responsibility of universities and educators in clinical placement settings (Schut et al. 2021). Telehealth placements during COVID‐19 pandemic restrictions rapidly increased as an innovative way to maintain student clinical practice opportunities during this time (McEvoy et al. 2024; Nahon et al. 2021). The rapid adoption of these types of placements prompted the development of resources to support educators to assess the development of these unique capabilities within these placements using the Assessment of Physiotherapy Practice, a performance‐based assessment commonly used within Australian clinical education (Nahon et al. 2021). However, since pandemic restrictions ended, many clinical placement experiences have largely returned to in‐person experiences.

Clinical education should be guided by the landscape of the healthcare system, and the needs of the future workforce (Bacopanos and Edgar 2015). Our findings demonstrate the wide use of telehealth delivery of physiotherapy management across the breadth of physiotherapy practice and delivered to patients across the lifespan. With the recognition that telehealth delivery remains an important part of contemporary physiotherapy practice, it remains vital that entry‐to‐ practice physiotherapy programs consider continued exposure to some telehealth delivery of services, and the opportunity to practice telehealth through clinical education (Martin, Mandrusiak, Russell, and Forbes 2022). Delivery of telehealth curriculum through the delivery of online modules and simulations has already been shown to be beneficial to physiotherapy students' perception of knowledge, skills and self‐efficacy aligned with telehealth capabilities (Martin, Mandrusiak, Lang, et al. 2022). However, further research aimed at improving the understanding of how and when physiotherapy students use telehealth in traditional clinical placement settings may be useful to inform curriculum changes.

Strengths of our study include the recency of our search strategy up to 2024, as well as capturing physiotherapy assessments and interventions delivered across the breadth of physiotherapy practice areas and populations. Although our search was current, it is possible we did not capture all physiotherapy assessments and interventions administered via synchronous telehealth, as we only included English studies and searched databases from 2013. Future research should consider exploring the best way to integrate telehealth education and training into entry‐to‐practice physiotherapy programs to prepare students for working in a digital environment.

In conclusion, the observed widespread and growing use of telehealth by physiotherapists highlights the importance of implementing telehealth education and training into entry‐to‐practice physiotherapy programs to prepare future physiotherapists with telehealth skills fit for contemporary practice.

## Author Contributions

**Luke M. Davies:** conceptualisation, methodology, investigation, data curation, formal analysis, writing–original draft, project administration, supervision. **Vidya Lawton:** data curation, review/editing–original draft. **Rebecca Bevan:** data curation, review/editing–original draft. **Mikayla Mestousis:** data curation, review/editing–original draft. **Verity Pacey:** methodology, investigation, data curation, formal analysis, review/editing–original draft, supervision.

## Ethics Statement

This scoping review did not involve the collection of primary data or participation of human subjects. All data included in the review were derived from publicly available sources; therefore, ethical approval was not required.

## Conflicts of Interest

The authors declare no conflicts of interest.

## Supporting information

Supporting Information S1

Supporting Information S2

Supporting Information S3

Supporting Information S4

Supporting Information S5

Supporting Information S6

## Data Availability

The data that supports the findings of this study are available in the supplementary material of this article.

## References

[msc70039-bib-0001] Albahrouh, S. I. , and A. J. Buabbas . 2021. “Physiotherapists' Perceptions of and Willingness to Use Telerehabilitation in Kuwait During the COVID‐19 Pandemic.” BMC Medical Informatics and Decision Making 21, no. 1: 122. 10.1186/s12911-021-01478-x. 33832473 PMC8028577

[msc70039-bib-0002] Bacopanos, E. , and S. Edgar . 2015. “Employment Patterns of Notre Dame Graduate Physiotherapists 2006–12: Targeting Areas of Workforce Need.” Australian Health Review 40, no. 2: 188–193. 10.1071/ah14244. 26254763

[msc70039-bib-0003] Bennell, K. L. , B. J. Lawford , B. Metcalf , et al. 2021. “Physiotherapists and Patients Report Positive Experiences Overall With Telehealth During the COVID‐19 Pandemic: A Mixed‐Methods Study.” Journal of Physiotherapy 67, no. 3: 201–209. 10.1016/j.jphys.2021.06.009. 34147399 PMC8188301

[msc70039-bib-0004] Bernhardsson, S. , A. Larsson , A. Bergenheim , et al. 2023. “Digital Physiotherapy Assessment vs Conventional Face‐to‐Face Physiotherapy Assessment of Patients With Musculoskeletal Disorders: A Systematic Review.” PLoS One 18, no. 3: e0283013. 10.1371/journal.pone.0283013. 36943857 PMC10030027

[msc70039-bib-0005] Chéron, M. , M. Ademi , F. Kraft , and H. Löffler‐Stastka . 2016. “Case‐Based Learning and Multiple Choice Questioning Methods Favored by Students.” BMC Medical Education 16: 1–7. 10.1186/s12909-016-0564-x. 26830675 PMC4736609

[msc70039-bib-0006] Cottrell, M. , C. L. Burns , A. Jones , et al. 2021. “Sustaining Allied Health Telehealth Services Beyond the Rapid Response to COVID‐19: Learning From Patient and Staff Experiences at a Large Quaternary Hospital.” Journal of Telemedicine and Telecare 27, no. 10: 615–624. 10.1177/1357633x211041517. 34726993 PMC8564219

[msc70039-bib-0007] Cottrell, M. A. , O. A. Galea , S. P. O’Leary , A. J. Hill , and T. G. Russell . 2017. “Real‐Time Telerehabilitation for the Treatment of Musculoskeletal Conditions Is Effective and Comparable to Standard Practice: A Systematic Review and Meta‐Analysis.” Clinical Rehabilitation 31, no. 5: 625–638. 10.1177/0269215516645148. 27141087

[msc70039-bib-0008] Davies, L. , R. S. Hinman , T. Russell , B. Lawford , M. Merolli , and K. L. Bennell . 2023. “Self‐Reported Confidence of Final Year Australian Physiotherapy Entry‐to‐Practice Students and Recent Graduates in Their Capability to Deliver Care via Videoconferencing.” European Journal of Physiotherapy 25, no. 6: 311–316. 10.1080/21679169.2022.2100928.

[msc70039-bib-0009] Davies, L. , B. Lawford , K. L. Bennell , T. Russell , and R. S. Hinman . 2023. “Telehealth Education and Training in Entry‐to‐Practice Physiotherapy Programs in Australian Universities: A Qualitative Study With University Educators.” Musculoskeletal Care 21, no. 2: 491–501. 10.1002/msc.1723. 36514306

[msc70039-bib-0010] Davies, L. , B. J. Lawford , and C. Chan . 2024. “Physiotherapy Students' Attitudes Toward the Use of Telehealth in Clinical Practice: A Cross‐Sectional Survey.” Health Science Reports 7, no. 4. 10.1002/hsr2.2067. PMC11033328 38650730

[msc70039-bib-0011] Dias, J. F. , V. C. Oliveira , P. R. T. Borges , et al. 2021. “Effectiveness of Exercises by Telerehabilitation on Pain, Physical Function and Quality of Life in People With Physical Disabilities: A Systematic Review of Randomised Controlled Trials With GRADE Recommendations.” British Journal of Sports Medicine 55, no. 3: 155–162. 10.1136/bjsports-2019-101375. 33060156

[msc70039-bib-0012] Eva, K. W. , G. Bordage , C. Campbell , et al. 2016. “Towards a Program of Assessment for Health Professionals: From Training Into Practice.” Advances in Health Sciences Education 21, no. 4: 897–913. 10.1007/s10459-015-9653-6. 26590984

[msc70039-bib-0013] Ezzat, A. M. , J.‐F. Esculier , S. L. Ferguson , C. Napier , and S. T. Wong . 2023. “Canadian Physiotherapists Integrate Virtual Care During the COVID‐19 Pandemic.” Physiotherapy Canada 75, no. 2: 134–145. 10.3138/ptc-2022-0092. 37736382 PMC10510558

[msc70039-bib-0014] Gava, V. , L. P. Ribeiro , R. P. G. Barreto , and P. R. Camargo . 2022. “Effectiveness of Physical Therapy Given by Telerehabilitation on Pain and Disability of Individuals With Shoulder Pain: A Systematic Review.” Clinical Rehabilitation 36, no. 6: 715–725. 10.1177/02692155221083496. 35230167

[msc70039-bib-0015] Govender, S. , and M. Mars . 2018. “The Perspectives of South African Academics Within the Disciplines of Health Sciences Regarding Telehealth and Its Potential Inclusion in Student Training.” African Journal of Health Professions Education 10, no. 1: 38–43. 10.7196/ajhpe.2018.v10i1.957.

[msc70039-bib-0016] Grant, C. M. , A. Jones , and H. Land . 2022. “Physiotherapists’ Perspectives on the Use of Telehealth for Service Delivery to Children With Developmental Delays: A Qualitative Focus Group Study.” Internet Journal of Allied Health Sciences and Practice 20, no. 2: 5. 10.46743/1540-580x/2022.2124.

[msc70039-bib-0017] Haines, K. J. , and S. Berney . 2020. “Physiotherapists During COVID‐19: Usual Business, in Unusual Times.” Journal of Physiotherapy 66, no. 2: 67–69. 10.1016/j.jphys.2020.03.012. 32291227 PMC7128648

[msc70039-bib-0018] Hawley‐Hague, H. , R. Lasrado , E. Martinez , E. Stanmore , and S. Tyson . 2023. “A Scoping Review of the Feasibility, Acceptability, and Effects of Physiotherapy Delivered Remotely.” Disability & Rehabilitation 45, no. 23: 3961–3977. 10.1080/09638288.2022.2138574. 36325612

[msc70039-bib-0019] Hirohama, K. , H. Tamura , K. Hamada , et al. 2023. “Effects of Non‐Face‐to‐Face and Noncontact Interventions on Knee Pain and Physical Activity in Older Adults With Knee Osteoarthritis: A Systematic Review and Meta‐Analysis.” Journal of Aging and Physical Activity 31, no. 2: 319–329. 10.1123/japa.2022-0037. 35961641

[msc70039-bib-0020] Hui, K. Y. , C. Haines , S. Bammann , et al. 2021. “To What Extent Is Telehealth Reported to be Incorporated Into Undergraduate and Postgraduate Allied Health Curricula: A Scoping Review.” PLoS One 16, no. 8: e0256425. 10.1371/journal.pone.0256425. 34411171 PMC8376028

[msc70039-bib-0021] MacDonald, C. W. , E. Lonnemann , S. M. Petersen , D. A. Rivett , P. G. Osmotherly , and J. M. Brismee . 2020. “COVID 19 and Manual Therapy: International Lessons and Perspectives on Current and Future Clinical Practice and Education.” Journal of Manual & Manipulative Therapy 28, no. 3: 134–145. 10.1080/10669817.2020.1782059. 32666912 PMC7480579

[msc70039-bib-0022] Martin, R. , A. Mandrusiak , R. Lang , T. Russell , and R. Forbes . 2022. “A Telehealth Curriculum: A Pre‐Post Study of Physiotherapy Students' Perceived Knowledge, Self‐Efficacy and Intentions for Future Use.” Focus on Health Professional Education: A Multi‐Professional Journal 23, no. 3: 56–72. 10.11157/fohpe.v23i3.595.

[msc70039-bib-0023] Martin, R. , A. Mandrusiak , T. Russell , and R. Forbes . 2022. “New‐Graduate Physiotherapists' Training Needs and Readiness for Telehealth.” Physiotherapy Theory and Practice 38, no. 13: 2788–2797. 10.1080/09593985.2021.1955423. 34282699

[msc70039-bib-0025] McEvoy, M. , C. Fryer , E. Ward , and S. Kumar . 2024. “Telehealth Usability in a University Student Physiotherapy Clinic During COVID‐19.” Musculoskeletal Care 22, no. 2: e1906. 10.1002/msc.1906. 38858804

[msc70039-bib-0026] Michell, A. , M. Besomi , P. Seron , et al. 2022. “Implementation of Physiotherapy Telerehabilitation Before and Post Covid‐19 Outbreak: A Comparative Narrative Between South American Countries and Australia.” Salud Pública de México 64: S31–S39. 10.21149/13160. 36130385

[msc70039-bib-0027] Moffatt, F. , and R. Kerry . 2018. “The Desire for ‘Hands‐on’ Therapy–A Critical Analysis of the Phenomenon of Touch.”

[msc70039-bib-0028] Nahon, I. , L. Jeffery , C. Peirsis , R. Dunwoodie , R. Corrigan , and A. Francis‐Cracknell . 2021. “Responding to Emerging Needs: Development of Adapted Performance Indicators for Physiotherapy Student Assessment in Telehealth.” Australian Journal of Clinical Education 9, no. 1: 17. 10.53300/001c.24960.

[msc70039-bib-0029] Peters, M. D. , C. Godfrey , P. McInerney , Z. Munn , A. C. Tricco , and H. Khalil . 2017. “Scoping Reviews.” Joanna Briggs Institute Reviewer’s Manual, 2015: 1–24.

[msc70039-bib-0030] Physiotherapy Board of Australia . 2023. “Physiotherapy Practice Thresholds in Australia & Aoetearoa New Zealnad for the Physiotherapy Profession.” https://www.physiotherapyboard.gov.au/Accreditation.aspx.

[msc70039-bib-0031] Rausch, A.‐K. , H. Baur , L. Reicherzer , et al. 2021. “Physiotherapists’ Use and Perceptions of Digital Remote Physiotherapy During COVID‐19 Lockdown in Switzerland: An Online Cross‐Sectional Survey.” Archives of physiotherapy 11: 1–10. 10.1186/s40945-021-00112-3. 34233763 PMC8261812

[msc70039-bib-0032] Reynolds, A. , N. Awan , and P. Gallagher . 2021. “Physiotherapists' Perspective of Telehealth During the Covid‐19 Pandemic.” International Journal of Medical Informatics 156: 104613. 10.1016/j.ijmedinf.2021.104613. 34688969 PMC8503965

[msc70039-bib-0033] Schut, S. , L. A. Maggio , S. Heeneman , J. Van Tartwijk , C. Van der Vleuten , and E. Driessen . 2021. “Where the Rubber Meets the Road—An Integrative Review of Programmatic Assessment in Health Care Professions Education.” Perspectives on Medical Education 10, no. 1: 6–13. 10.1007/s40037-020-00625-w. 33085060 PMC7809087

[msc70039-bib-0034] Seron, P. , M.‐J. Oliveros , R. Gutierrez‐Arias , et al. 2021. “Effectiveness of Telerehabilitation in Physical Therapy: A Rapid Overview.” Physical Therapy 101, no. 6: pzab053. 10.1093/ptj/pzab053. 33561280 PMC7928601

[msc70039-bib-0035] Shaw, D. K. 2009. “Overview of Telehealth and its Application to Cardiopulmonary Physical Therapy.” Cardiopulmonary Physical Therapy Journal 20, no. 2: 13–18. 10.1097/01823246-200920020-00003. PMC2845264 20467533

[msc70039-bib-0036] Suso‐Marti, L. , R. La Touche , A. Herranz‐Gomez , S. Angulo‐Diaz‐Parreno , A. Paris‐Alemany , and F. Cuenca‐Martinez . 2021. “Effectiveness of Telerehabilitation in Physical Therapist Practice: An Umbrella and Mapping Review With Meta‐Meta‐Analysis.” Physical Therapy 101, no. 5. 10.1093/ptj/pzab075. PMC7928612 33611598

[msc70039-bib-0037] Telehealth Services Australia . 2020. “What Is Telehealth.” https://telehealthservices.com.au/about/what‐is‐telehealth/.

[msc70039-bib-0038] Tricco, A. C. , E. Lillie , W. Zarin , et al. 2018. “PRISMA Extension for Scoping Reviews (PRISMA‐ScR): Checklist and Explanation.” Annals of Internal Medicine 169, no. 7: 467–473. 10.7326/m18-0850. 30178033

[msc70039-bib-0039] Tsai, L. L. Y. , R. J. McNamara , C. Moddel , J. A. Alison , D. K. McKenzie , and Z. J. McKeough . 2017. “Home‐Based Telerehabilitation via Real‐Time Videoconferencing Improves Endurance Exercise Capacity in Patients With COPD: The Randomized Controlled TeleR Study.” Respirology (Carlton, Vic.) 22, no. 4: 699–707. 10.1111/resp.12966. 27992099

[msc70039-bib-0040] van Egmond, M. A. , M. van der Schaaf , T. Vredeveld , et al. 2018. “Effectiveness of Physiotherapy With Telerehabilitation in Surgical Patients: A Systematic Review and Meta‐Analysis.” Physiotherapy 104, no. 3: 277–298. 10.1016/j.physio.2018.04.004. 30030037

[msc70039-bib-0041] World Confederation for Physiotherapy, and International Network of Physiotherapy Regulatory Authorities . 2020. “Report of the WCPT/INPTRA Digital Physical Therapy Practice Task Force.” https://world.physio/sites/default/files/2020‐06/WCPT‐INPTRA‐Digital‐Physical‐Therapy‐Practice‐Task‐force‐March2020.pdf.

[msc70039-bib-0042] World Physiotherapy . 2021. “Physiotherapist Education Framework.” https://world.physio/sites/default/files/2021‐07/Physiotherapist‐education‐framework‐FINAL.pdf.

[msc70039-bib-0043] Zischke, C. , V. Simas , W. Hing , N. Milne , A. Spittle , and R. Pope . 2021. “The Utility of Physiotherapy Assessments Delivered by Telehealth: A Systematic Review.” Journal of global health 11: 04072. 10.7189/jogh.11.04072. 34956637 PMC8684795

