# Supplementary material for: Informing the Development of Telehealth Education in Physiotherapy Programs. Assessments and Interventions for Individuals Accessing Physiotherapy Care via Synchronous Telehealth. A Scoping Review
Source: Musculoskeletal Care. 2025 Jan 9;23(1):e70039. doi: 10.1002/msc.70039 (PMC11717065; doi:10.1002/msc.70039)
Supplement: Supplementary file 1 — Supporting Information S1 [file MSC-23-e70039-s002.docx]

| **Author,**  Appendix A. Summary table of included studies  **Year** | **Country** | **Study type** | **Participants (Sample size, Age in years)** | **Healthcare service/providers** | **Condition** | **Assessment** | **Intervention** | **Mode/s of delivery** | **Telehealth technology** | **Platform** |
| --- | --- | --- | --- | --- | --- | --- | --- | --- | --- | --- |
| Ahikari et al., 2020^1^ | Nepal | Retrospective | n=15 Age: range 36 - 70 | Physiotherapist | Prolapse intervertebral disc, tennis elbow, rheumatoid arthritis, mechanical low back pain, traumatic ankle sprain, neck pain | NA | Exercise Education Coaching | Synchronous | Telephone | NA |
| Aktan et al., 2023^2^ | Turkey | Interrater reliability | n=50 Age: mean 54.5; SD 6.3 | Physiotherapists with more than 7 years experience | Type 2 diabetes mellitus | 30 second sit-to-stand | Exercise | Synchronous | Telephone | WhatsApp (VC) |
| Aloraini, Saleh et al., 2023^3^ | NR | Pilot feasibility | n=40 Age: Mean 63.25; SD 10.82 | Physiotherapist with 14 years of working with musculoskeletal impairments | Knee Osteoarthritis | NA | Exercise Education | Synchronous | Telephone | NA |
| Alsobayel et al., 2021^4^ | Saudi Arabia | Prospective quasi-experimental pre–post intervention repeated measure design | n=95 Age: mean 33; SD 8 | Physiotherapist | Non-urgent musculoskeletal condition that was deemed appropriate for non-surgical management | NA | Exercise Education | Synchronous | VC | Google Meet |
| Anghelescu, 2022^5^ | Romania | Prospective | n=17 Age: mean 65.9; SD 4.87 | Physiotherapist | Idiopathic Parkinsons Disease | NA | Exercise Coaching | Synchronous | VC & Telephone | Google meet, Skype, Whatsapp |
| Baehr et al., 2024^6^ | United States of America | Parallel mixed methods design | n=22 Age: range 26-68 | Physiotherapist with adapted exercise teaching experience | Spinal cord injury (complete or incomplete) | NA | Exercise Coaching | Synchronous | VC | Zoom |
| Baehr et al., 2023^7^ | United States of America | Feasibility | n=11 Age: mean 49.5; SD 16.7 | A licensed physiotherapist with extensive experience teaching adapted exercise both F2F and virtually | Spinal cord injury | NA | Exercise Coaching | Synchronous | VC | Zoom |
| Batalik et al., 2021^8^ | Czech Republic |  | n=19 Age: mean 60.4; SD 9.6 | Physiotherapist | Coronary heart disease | NA | Exercise Coaching | Synchronous | Telephone | NA |
| Batsis et al., 2021^9^ | United States of America | Single arm intervention | n=44 Age: mean 73.2; SD 3.9 | Physiotherapist | Obesity | NA | Exercise Coaching | Hybrid Synchronous  Every 3-4 weeks 1 session was F2F | VC | Zoom |
| Bennell et al., 2022^10^ | Australia | RCT | n=347 Age: mean 64.7; SD 8.16 | Physiotherapist | Knee Osteoarthritis | NA | Exercise Education Coaching | Synchronous | VC | Zoom |
| Benz, 2023^11^ | Australia | Retrospective chart audit | n=30 Age: median14; IQR 10 - 16 | Physiotherapist from Hospital in the Home service | Cystic fibrosis | NA | Exercise | Hybrid - Synchronous and F2F | NR | NR |
| Beresford and Norwood, 2022^12^ | United States of America | Retrospective observational, longitudinal | n=814 Age: 40.85; SD 11.89 | Experienced Physiotherapists (average 10 years), and more than 60% were board-certified in orthopaedic, sports, and women’s health | Musculoskeletal condition such as low back pain, neck pain, arthritis, sprains, strains, or similar overuse injuries | Subjective assessment and objective examination | NA | Synchronous | VC | Omada Health |
| Bernocchi et al., 2016^13^ | Italy | Pilot study | n=26 Age: mean 70; SD 10 | Physiotherapist | Post stroke patients | NA | Exercise | Hybrid - initial visit at patients home, subsequent consults through VC | VC | TelereRiab, |
| Bernocchi et al., 2018^14^ | Italy | RCT | n=50 Age: mean 71; SD 9 | Physiotherapist | Combine chronic obstructive pulmonary disorder and chronic heart failure | NA | Education Coaching | Synchronous | Telephone | NA |
| Bickton et al., 2021^15^ | Malawi | Case report | n=1 Age: 46 | Physiotherapist certified in pulmonary rehabilitation | Post acute severe Covid-19 | Patient- reported subjective assessments | Exercise Education | Synchronous | Telephone and VC | WhatsApp (videoconference) |
| Blioumpa et al., 2023^16^ | Greece | Randomised parallel pilot study | n=11 Age: mean 60.3; SD 9.3 | Physiotherapist | Type 2 diabetes mellitus | NA | Exercise | Hybrid - first educational session at university facilities.  Synchronous intervention | VC | Skype |
| Bowman et al., 2023^17^ | Australia | Cross-sectional observational | n=30 Age: median 62.5; IQR: 51.8-66.5 | Physiotherapist and Exercise Physiologist | Oncology | 30 Second sit to stand | NA | Synchronous | VC | NR |
| Brennan et al., 2022^18^ | Ireland | Mixed method feasibility | n=12 Age: mean 65.42; SD 7.24 | Physiotherapist | Esophago-gastric cancer | NA | Exercise | Synchronous | VC | Digital Therapeutics Platform created by Salaso Health Solutions Ltd. |
| Brennen et al., 2023^19^ | Australia | Pre-post single group feasibility clinical trial | n=36 Age: median 58; IQR 17 | Physiotherapist with postgraduate qualifications and 16 years’ clinical experience in pelvic floor physiotherapy | Endometrial, uterine, cervical or ovarian cancer (stages I–III) | NA | Exercise Coaching Education | Synchronous | VC | Zoom |
| Brouwers et al., 2021^20^ | Netherlands | Prospective randomized clinical trial | n=153 Age: Mean 60.7; SD 9.5 | Physiotherapist | Coronary artery disease | NA | Exercise | Synchronous | VC | NR |
| Bulguroglu and Bulguroglu, 2023^21^ | Turkey | RCT | n=20 Age: median 28; IQR: 25-31 | Pilates and Physiotherapy Institute certified and experienced Ph.D. Physiotherapist | Healthy university workers | NA | Exercise | Synchronous | VC | Microsoft Teams |
| Campbell et al., 2022^22^ | United States of America | Cross-sectional | n=17 Age: mean 38.34; SD 12.71 | Physiotherapist | Mild traumatic brain injury | NA | Exercise | Synchronous | VC | Webex |
| Cheville et al., 2019^23^ | United States of America | Randomised 3-arm trial | n=344 Age: mean 65.4; SD 11.3 | Physiotherapists specialised in cancer rehab, within Multidisciplinary team (nurses and physicians) | Pathologically confirmed stage IIIC or solid IV or haematological cancer | Screening for physical impairments | Exercise Education | Synchronous | Telephone | NA |
| Colombage et al., 2023^24^ | Australia | Pre-post trial | n=54 Age: mean 50; SD7.3 | Physiotherapist with postgraduate pelvic floor training | Breast cancer and stress incontinence | NA | Exercise Coaching | Hybrid - synchronous and asynchronous | VC | Zoom |
| Conlan et al., 2016^25^ | Australia | Case series | n=6 Age: mean 36.5; SD 11.5 | Physiotherapist | Stress incontinence | Subjective assessment | Exercise Education | Synchronous | VC | Skype |
| Cooley Hidecker et al., 2022^26^ | United States of America | Prospective cohort study | n=15 Age: mean 73.3; range 57 - 93 | Physiotherapist experienced treating patients with Parkinsons disease, within MDT (speech therapy, pharmaceutical care, nurse) | Parkinsons disease | NA | Exercise | Hybrid - synchronous and F2F | VC | Zoom |
| Coronado, Rogelio et al., 2021^27^ | United States of America | Case series | n=8 Age: mean 53.4; SD 14.9 | Physiotherapist with 15 years experience with patients with musculoskeletal pain | Post anterior cervical discectomy and fusion | NA | Exercise Coaching | Synchronous | Telephone | NA |
| Coronado, Rogelio et al., 2020^28^ | United States of America | Pilot study | n=8 Age: mean 20.12; SD 2.58 | Physiotherapist | Anterior cruciate ligament | NA | Exercise Coaching | Synchronous | Telephone | NA |
| Cottrell et al., 2018^29^ | Australia | Inter-rater agreement study | n=42 Age: mean 52.7; SD 14.5 | Physiotherapists with post-graduate qualifications in musculoskeletal physiotherapy and at least 2 year sin advanced practice role | Musculoskeletal complaints to lumbar spine, knee or shoulder | Triage, Pragmatic assessment based on presentation and ability to safely and adequately complete clinical tests | N/A | Synchronous | VC | eHab |
| Cronin et al., 2022^30^ | Ireland | Case study | n=1 Age:68 | Physiotherapist with 8 years stroke rehabilitation and 5 years Pilates instructor experience | Left basal ganglia infarct >6/52 post injury and discharged from rehabilitation | NA | Exercise | Synchronous | VC | Zoom |
| de la Plaza San Frutos et al., 2023^31^ | Spain | Prospective longitudinal non-randomised study | n=50 Age: median 49; IQR 38 - 55.75 | Specialised respiratory physiotherapist with more than 10 years experience | Post infective COVID-19 | Heart rate, respiratory rate and oxygen saturation, Mahlers dyspnoea index | Exercise | Synchronous | VC | Zoom |
| Duruturk and Ozkoslu, 2019^32^ | Turkey | RCT | n=23 Age: mean 52.82; SD 11.86 | Physiotherapist | Type 2 diabetes mellitus | NA | Exercise Coaching | Hybrid- first session, then Synchronous. | VC | NR |
| Ekmekyapar et al., 2023^33^ | Belgium | Prospective, longitudinal non- randomised study | n=15 Age: mean 63.13; SD 9.89 | Physiotherapist | Parkinsons disease | N/A | Exercise | Hybrid- F2F and synchronous | VC | NR |
| Eldemir et al., 2023^34^ | Turkey | Randomized evaluator-blind trial. | n=15 Age: mean 57.87; SD 9.79 | Physiotherapist with rehabilitation experience in Parkinsons disease for at least 4 years. | Parkinsons disease | NA | Exercise | Synchronous | VC | NR |
| Eyuboglue et al., 2023^35^ | Turkey | RCT | n=22 Age: mean 44.36; SD 8.56 | Physiotherapist with an instructor certificate in yoga | Obstructive sleep apnoea syndrome | NA | Exercise | Synchronous | VC | Zoom |
| Filakova et al., 2023^36^ | Czech Republic | Prospective single-arm interventional study | n=11 Age: mean 60.3; SD 10.2 | Physiotherapist specializing in cardiac rehabilitation for more than 5 years | Lymphoma | NA | Exercise Coaching | Hybrid - synchronous and asynchronous | Telephone and web-based platform | Polar flow |
| Flynn et al., 2021^37^ | Australia | Feasibility RCT | n=20 Age: mean 72; SD 7.3 | Physiotherapists | Parkinsons disease | NA | Exercise | Hybrid- F2F and synchronous | Telephone | NA |
| Gagnon et al., 2021^38^ | Canada | Prospective interventional single-cohort pilot study | n=7 Age: median 16.9; range 11.3- 20.8 | Physiotherapist | Arthrogryposis multiplex congenita | ROM measurements and mobility, pain assessment using the Adolescent and Paediatric Pain Tool (APPT). | Exercise | Hybrid- synchronous and asynchronous | VC and web- based platform | Zoom, Polar Flow |
| Galiano-Castillo et al., 2014^39^ | Spain | Descriptive crossover design | n=30 Age: mean 46.33; SD 9.05 | Physiotherapist with >4 years experience in cancer care | Lymphoedema | Arm measurements using a tape measure | NA | Hybrid- F2F and synchronous | VC | Wormhole Web Conference and Skype software |
| Gavazzi et al., 2021^40^ | United States of America | Interrater reliability study design | n=21 Age: mean 10.1; SD 11.0 | Physiotherapists who had extensive experience using the GMFM-88 | leukodystrophy | GMFM-88 | NA | Synchronous | VC | Cisco WebEx |
| Gerguz and Aras Bayram, 2023^41^ | Turkey | RCT | n=20 Age: mean 12.10; SD 3.54 | Physiotherapist with a yoga instructor certificate | fit and healthy young athletes (tennis players) | NA | Exercise | Synchronous | VC | Zoom |
| Gillespie et al., 2021^42^ | Canada | Interrater reliability study design | n=20 Age: mean 72; SD 9.4 | Physiotherapist rater had administered the BBS frequently in practice (>5 y), and 2–5 years of experience working in community stroke rehabilitation | stroke | BBS | NA | Hybrid- F2F and synchronous | VC | Skype for Business videoconferencing software |
| Goode et al., 2018^43^ | United States of America | Pilot feasibility trial | n=60 Age: Mean 69.5; SD 3.7 | Physiotherapist | chronic Low back pain | NA | Exercise | Synchronous- 3 telephone follow ups | Telephone | NA |
| Gutiérrez et al., 2013^44^ | Spain | RCT | n=24 Age: mean 39.69; SD 8.13 | Physiotherapist | multiple sclerosis | NA | Exercise | Synchronous | VC | NR |
| Hamidfar et al., 2023^45^ | France | Multicentre prospective, pilot study | n=21 Age: mean 53; SD 18 | Respiratory physiotherapist who was an expert on the device | non-cystic fibrosis bronchiectasis | NA | Exercise  Coaching | Synchronous- telecare session every 10 | VC | SIMEOX web application |
| Hinman et al., 2017^46^ | Australia | Qualitative | n=12 Age: mean 62; SD 7 | Physiotherapists | Osteoarthritis | NA | Exercise | synchronous | VC | Skype for Business |
| Hinman et al., 2020^47^ | Australia | RCT | n=87 Age: mean 62.4; SD 9.1 | Physiotherapist trained in behaviour change | Osteoarthritis | Average knee pain measured via numeric rating scale, Functional status measured by the Western Ontario and McMaster Universities Osteoarthritis Index28 | Exercise Coaching | Hybrid- asynchronous assessment (electronic or paper-based) with synchronous telephone based intervention | Telephone | NA |
| Holland et al., 2013^48^ | Australia | Single- cohort pilot study | n=8 Age: range 56- 83 | Experienced physiotherapist | Chronic obstructive pulmonary disorder | NA | Exercise | Synchronous | VC | VSee |
| Hong et al., 2022^49^ | United States of America | Observational, prospective cohort study | n=75 Age: mean 46.8; SD 11.1 | Physiotherapist | acute Musculoskeletal conditions | Functional assessment | Exercise | Synchronous | VC | NR |
| Horton et al., 2021^50^ | United States of America | Matched-cohort design | n=17 Age: mean 33.8; SD 10.3 | Physiotherapist | Femoral acetabular impingement | NA | Exercise Coaching | Hybrid F2F Synchronous | NR | American Well platform |
| Huzmell et al., 2022^51^ | Turkey | Non-randomised study | n=7 Age: mean 46; SD 11.37 | Physiotherapist | healthy, sedentary women | NA | Exercise | synchronous | Telephone | Skype for Business |
| Hwang et al., 2017^52^ | Australia | Randomised parallel non-inferiority | n=53 Age: mean 68; SD 14 | Physiotherapists highly experienced in exercise for chronic heart failure patients | Chronic heart failure | N/A | Exercise Education | Synchronous | VC | Adobe Connect |
| Janela et al., 2024^53^ | United States of America | Prospective cohort | n=326 Age: mean 44.8; SD 9.1 | Physiotherapist specialising in pelvic health | Urinary incontinence | Subjective assessment | Exercise Education | Hybrid - asynchronous and synchronous | VC | NR |
| Jasper et al., 2023^54^ | United States of America | Reliability study | n=6 Age: mean 65.5; SD 3.67 | Physiotherapists | Community dwelling older adults with orthopaedic or cardiac issues | 2 minute step test, 30sec chair stand test, timed up and go test | NA | Synchronous | VC | Zoom |
| Kamali et al., 2023^55^ | Turkey | RCT | n=27 Age: mean 46.29; SD 9.51 | Physiotherapists | Stress urinary incontinence | NA | Exercise Coaching | Synchronous | VC | NR |
| Kenis-Coskun, et al, 2022^56^ | NR | RCT | n=10 Age: mean 8.8; SD 2.93 | Physiotherapist | Duchenne's muscular dystrophy (ambulatory) | NA | Exercise | Synchronous | VC | NR |
| Kepenek-Varol et al, 2022^57^ | NR | RCT | n=25 Age: mean 31.3; SD 11.4 | Physiotherapist | Not exposed to COVID-19 | NA | Exercise Education | Synchronous | Telephone | WhatsApp |
| Kinder et al, 2019^58^ | NR | Case report | n=3 Age: 41, 39, 55 | Physiotherapist | Premature ejaculation, diastasis recti, maintenance of stress urinary incontinence | Assessment of abdominal curl-up and intra-abdominal  pressure control | Exercise Education | Hybrid - Initial F2F, follow up consultations via synchronous | VC | BlueJay Engage |
| Kraal et al., 2014^59^ | Netherlands | RCT | n=25 Age: mean 60.6; SD 7.5 | Physiotherapist | Post myocardial infarction, unstable angina or revascularisation procedure with low to moderate risk of future cardiac events | NA | Education Coaching | Hybrid - outpatient F2F, asynchronous and synchronous | Telephone | NA |
| Kratz et al, 2020^60^ | United State of America | RCT | n=10 Age: mean 45.9; SD 8.0 | Physiotherapist | Multiple sclerosis | NA | Exercise Education | Hybrid - F2F, asynchronous and synchronous | Telephone | NA |
| Lafaro et al., 2020^61^ | United States of America | Prospective cohort study | n=34 Age: median 73; range 66 - 84 | Physiotherapist and occupational therapist | Pre- and post- gastrointestinal and lung cancer procedures | NA | Coaching | Hybrid - F2F and synchronous | VC | Zoom |
| Lavoie et al., 2021^62^ | Canada | Pilot pre- post- design | n=11 Age: mean 69.2; SD 3.6 | Physiotherapist | Parkinsons disease | NA | Exercise Education | Synchronous | VC | Teraplus |
| Lawford et al., 2018^63^ | Australia | A qualitative study embedded within a RCT | n=20 Age: mean 59; SD 9 | Physiotherapists, trained in behaviour change support | OA | NA | Exercise | Hybrid- synchronous and asynchronous (information booklet) | Telephone | NA |
| Lawford et al., 2022^64^ | Australia | A prospective within-participant repeated-measure design | n=57 Age: mean 63.1; SD 9.3 | Physiotherapists with at least 1 year of clinical experience | chronic lower limb musculoskeletal pain | Various performance-based tests | NA | Hybrid- F2F and synchronous | VC | Zoom Video Communications, Inc., San Jose, CA |
| Lawford et al., 2020^65^ | Australia | Longitudinal study | n=84 Age: mean 62.3; SD 9.3 | Physiotherapists with two years musculoskeletal experience | Osteoarthritis | NA | Education Coaching | Synchronous | Telephone | NA |
| Lawford et al., 2023^66^ | Australia | 3-arm RCT | n=300 Age: mean 65; SD 8.2 | Physiotherapist | Osteoarthritis | NA | Education | Hybrid- synchronous and asynchronous (booklets, ex instructions) | VC | Zoom Video Communications Inc |
| Le Berre et al., 2023^67^ | Canada | Non-inferiority RCT | n= 34 Age: mean 69; SD 6 | Experienced pelvic floor physiotherapist | Urinary incontinence | NA | Exercise Coaching Education | Hybrid- Synchronous and asynchronous | VC | Zoom |
| Lee et al., 2023^68^ | Korea | Prospective comparative study | n=31 Age: mean 26.96; SD 5.99 | Physiotherapist | Patellofemoral pain syndrome | NA | Exercise | Hybrid- synchronous and asynchronous | Telephone | NA |
| Lee et al., 2022^69^ | Korea | RCT | n=30 Age: mean 25.64; SD 3.18 | Physiotherapist | rounded shoulder posture (otherwise healthy) | NA | Exercise Education | Hybrid- synchronous and asynchronous | VC | NR |
| Lotan et al., 2021^70^ | Ireland | Pilot study | n=5 Age: mean 9; SD 5.61 | Physiotherapist | Rett syndrome | NA | Exercise | synchronous telehealth | VC | Skype |
| Lundgren et al., 2023^71^ | Norway | Prospective RCT | n=26 Age:≥18 | Experienced physiotherapist specialised in cardiac rehabilitation | Chronic heart failure | NA | Exercise | synchronous telehealth | VC | NR |
| Mani et al., 2021^72^ | Malaysia | Validity/Reliability | n=11 Age: mean 32.7; SD 10.9 | Physiotherapists with more than 5 years clinical experience in neck pain. | Non-specific neck pain | ROM, muscular endurance testing and postural assessment. | NA | synchronous telehealth | VC | TelePTsys |
| Mantelatto Andrade et al., 2024^73^ | Brazil | Cohort study | n=33 Age: mean 13.4; SD 1.3 | Physiotherapist | Idiopathic scoliosis | Radiological assessment | Exercise Education | Hybrid, synchronous and asynchronous telehealth | Telephone and VC | WhatsApp video |
| Manzak Dursun et al., 2024^74^ | Turkey | RCT | n=16 Age: NR (children and adolescents) | Physiotherapist | Idiopathic scoliosis | NA | Exercise Coaching | Hybrid synchronous and asynchronous telehealth. And, hybrid F2F and telehealth | VC | NR |
| Martin et al., 2021^75^ | Belgium | Prospective observational study | n=14 Age: mean 60.8; SD 10.4 | Experienced physiotherapist involved in the pulmonary rehabilitation program | Covid-19 | One minute sit-to-stand test | Exercise | Synchronous telehealth | VC | Teams |
| Master et al., 2024^76^ | NR | Feasibility RCT | n=8 Age: mean 65.4; SD 15.7 | Physiotherapist with 15 years experience delivering remote behavioural interventions | Patients undergoing laminectomy for a lumbar degenerative condition | Weekly walking goal (pedometer) | Coaching | Hybrid synchronous and asynchronous telehealth | VC | Zoom |
| Mehta et al., 2021^77^ | United States of America | Reliability study | n=54 Age: mean 24.5; SD 1.9 | Physiotherapist with over 20 years’ experience. | Healthy adults | ROM of knee and wrist | NA | Synchronous telehealth | Virtual camera system | NR |
| Mesa-Castrillon et al., 2024^78^ | Australia | RCT | n=60 Age: mean 63.27; SD 11.4 | Physiotherapists with at least two years of clinical experience in Australia | Chronic non-specific low back pain or knee osteoarthritis | NA | Exercise | Synchronous and asynchronous telehealth | VC | Physitrack |
| Middleton et al., 2020^79^ | United States of America | Pilot case study | n=1 Age: 67 | Physiotherapist with more than five years of clinical experience | Stroke, hypertension, diabetes mellitus | Various functional tests and patient reported scales | Exercise | Synchronous telehealth only | VC | Vidyo |
| Mobbs et al, 2022^80^ | Australia | RCT | n=7 Age Less than 4 months corrected age | Physiotherapists | Premature birth | NA | Exercise Education | Hybrid F2F and synchronous telehealth | NR | PreEMPT |
| Nambi et al., 2023^81^ | Saudi Arabia | RCT | n=64 Age: mean 48.6; SD 4.2 | Three physiotherapists with up to fifteen years of clinical experience treating type II diabetes mellitus | Type II diabetes mellitus | NA | Exercise | Hybrid F2F and telehealth. Synchronous telehealth | VC | NR |
| Nelson et al., 2020^82^ | Australia | RCT | n=35 Age: mean 62; SD 9 | Physiotherapists | Post operative total hip replacement | NA | Exercise | Synchronous and asynchronous telehealth | VC | eHAB |
| Nicola et al., 2018^83^ | Australia | Test, retest | n=59 Age: range 5 - 11 | Physiotherapists. Three paediatric physiotherapists and two physiotherapy students. | Typically developing children | Movement Assessment Battery for Children. | NA | Synchronous telehealth | VC | eHAB |
| Nunez-Cortes et al., 2023^84^ | Spain | RCT | n=25 Age: Mean 44.7; SD 7.74 | Physiotherapist with 7 years of experience in pain neuroscience education. | Carpal tunnel syndrome | NA | Exercise Education | Synchronous | Telephone | WhatsApp (videoconference) |
| O'Neil et al., 2023^85^ | Canada | Convergent mixed-method approach with an alternating single-subject design | n=5 Age: range 21-60 | Physiotherapist with over 11 years of clinical experience, over 7 years of telerehabilitation experience. | Moderate to severe TBI | NA | Exercise | Synchronous | VC | NR |
| Okpara et al., 2023^86^ | Canada | Parallel group feasibility RCT | n=35 Age: Mean 78.2; SD 7 | Physiotherapists | Vulnerable older adults with frailty | NA | Exercise | Synchronous | VC | Zoom |
| Okudan et al., 2024^87^ | Turkey | RCT | n=30 Age: mean 52.27; SD 45-60 | 7-year experienced physiotherapist | Low back pain caused by facet joint arthrosis | Tele-assessment appointment by phone with a physiotherapist to establish baseline measures | Exercise | Synchronous | VC | Google Meets |
| Onan et al., 2023^88^ | Turkey | RCT | n=15 Age: mean 37.4; SD 10.58 | Physiotherapist holding Master of Science Degree | Chronic neck pain | Functional capacity assessment including adaptation of the Neck Functional Capacity Evaluation Test | Exercise | Synchronous | VC | NR |
| Ozlu et al., 2024^89^ | Turkey | RCT | n=40 Age: mean 36.20; SD 6.61 | Physiotherapist with at least 3 years of experience | Chronic non-specific neck pain | NA | Exercise | Synchronous | VC | NR |
| Ozturk et al., 2022^90^ | Turkey | NR | n=21 Age: mean 41.05; SD 12.64 | Experienced physiotherapist | Overweight and obese | Senior Fitness Test protocol | Exercise | Synchronous | VC | NR |
| Palmcrantz et al., 2017^91^ | Sweden | Feasibility study | n=15 Age: Mean 66; SD 16 | Physiotherapists specialising in stroke rehabilitation | Stroke patients | NA | Exercise Coaching | Synchronous | VC | DISKO-tool |
| Paolucci et al., 2022^92^ | Italy | NR | n=28 Age: mean 56.61; SD 8.56 | Physiotherapist MDT including physiatrist and psychologist were present with cameras off | Fibromyalgia | NA | Exercise Coaching | Synchronous | VC | Zoom |
| Park et al., 2024^93^ | Canada | Feasibility Study | n=32 Age: median 64.5; Quartile 1 51; Quartile 2 72 | Physiotherapist who completed a 3-hour training program | Post stroke | Functional Comorbidity Index, Modified Rankin Scale, National Institutes of Health (NIH) Stroke Severity Scale Timed “Up & Go” test | Exercise Education | Synchronous | VC | NR |
| Pastana Ramos et al., 2023^94^ | Brazil | RCT | n=8 Age: Mean 60.7; SD 49–72 | Physiotherapist | Parkinsons Disease | NA | Exercise Coaching | Synchronous | VC | NR |
| Pehlivan et al., 2022^95^ | Turkey | RCT | n=17 Age: median 50.76; range 32-82 | Physiotherapist | COVID-19 | Modified Medical Research Council dyspnea score, sit‑to‑stand test and short physical performance battery, Saint George Respiratory Questionnaire and Beck Depression Inventory. | Exercise Education | Synchronous | VC | NR |
| Peterson, 2018^96^ | United States of America | Case series | n=3 Age: 21, 47 and 67 | Physical therapist was a board-certified orthopaedic clinical specialist and had 12 months of mentoring in the use of telerehabilitation | Chronic lower back pain | NA | Education | Hybrid - asynchronous and synchronous | VC | Zoom |
| Peterson, 2023^97^ | United States of America | Case series | n=3 Age: 65, 30, 72 | Physiotherapist working at a private outpatient office | Lower back pain and cervicogenic headaches | ROM testing, muscular endurance and strength testing, self-administered sensation testing and hip mobility testing | Exercise Education | Hybrid - Synchronous F2F, asynchronous | VC and Telephone | Doxy.me |
| Petter Rodrigues et al., 2023^98^ | Brazil | Pre-post study | n=40 Age: Mean 56.5; SD 12.0 | Physiotherapist with expertise in pelvic floor dysfunction | Urinary incontinence | NA | Exercise Coaching | Hybrid - Asynchronous and Synchronous | VC | WhatsApp (videoconference) |
| Pinto et al., 2023^99^ | Brazil | Feasibility clinical trial (pre-post) | n=12 with PD, 14 without PD.  Age: Mean 69 | Professional dancer and physiotherapist with 25 years of dance practice and 5 years of clinical practice as a physiotherapist with experience with Parkinsons Disease. | Older Adults With and Without Parkinson’s Disease | Five times sit to stand test | Exercise | Hybrid - Asynchronous and Synchronous | VC | Zoom |
| Piraux et al., 2019^100^ | Belgium | Pilot randomised study | n=9 Age: mean 47.2; SD  10.6 | Physiotherapist | Human immunodeficiency virus | NA | Exercise Coaching | Hybrid - asynchronous and synchronous telehealth | Telephone | NR |
| Plaza et al., 2023^101^ | Australia | Randomized, controlled, noninferiority clinical pilot trial | n=23 Age: mean 48.4; SD 14.2 | Physiotherapist with burn experience | Burn injuries | NA | Exercise | Hybrid - Synchronous, asynchronous and F2F | VC | eHAB |
| Pleguezuelos et al., 2024^102^ | Spain | RCT | n=66 Age: mean 54.6; SD 11.7 | Experienced physiotherapist with more than 4 years of clinical experience in chronic diseases. | Post-COVID-19 sequelae | NA | Exercise | Synchronous | VC | NR |
| Post et al., 2023^103^ | United States of America | Secondary Analysis of RCT impacted by COVID lockdowns | n=41 (Telehealth only =20, hybrid=21) Age: Mean 43.35; SD 15.47 | Experienced physiotherapist of 7 years, with additional training as an orthopaedic clinical specialist | Chronic Achilles Tendinopathy | Movement evoked pain on heel raises, maximum number of heel raises | Exercise Education | Hybrid - synchronous, asynchronous and F2F AND synchronous and asynchronous | VC | Zoom |
| Ramachandra & Preetha, 2022^104^ | India | Case Report | n=1 Age: 29 | Physiotherapist | Pelvic girdle dysfunction in pregnancy | Examination of posture, gait and movement to evoke pain, tenderness on palpation | Exercise Education | Synchronous | VC | WhatsApp |
| Richardson et al., 2017^105^ | Australia | Repeated-measures design | n=18 Age: mean 23; SD 7 | Physiotherapist | Knee pain | self-palpation, self-applied modified orthopaedic tests including manual muscle tests, active movements and functional tasks. | NR | Synchronous | VC | eHAB |
| Rodriguez-Blanco et al., 2023^106^ | Spain | RCT | n=24 Age: mean 38.75; SD 15.40 | Physiotherapist | Non-Hospitalized Post-COVID-19 with long COVID symptoms | Six minute walk test, Thirty second sit to stand test | Exercise | Synchronous | VC | Whatsapp |
| Rodriguez-Blanco et al., 2022^107^ | Spain | RCT | n=55 Age: Mean 38.56; SD 11.46 | Physiotherapist | Acute COVID-19 with <1 week of symptoms | Six minute walk test, Thirty second sit to stand test | Exercise | Synchronous | VC | Whatsapp |
| Rosenbek Minet et al., 2015^108^ | Denmark | Prospective cohort study | n=37 Age: Mean 69.2; SD 8.8 | Physiotherapist and Occupational Therapist | Severe chronic obstructive pulmonary disease post discharge form hospital for exacerbation | NA | Exercise Coaching | Synchronous | VC | Bespoke closed secure system |
| Russell et al., 2023^109^ | Australia | Cross-sectional observational study | n=27 Age: range 18 - over 61 | Single physiotherapist experienced in haemophilia care and audioconferencing/videoconferencing consultations | Adults with inherited bleeding disorders | Individualised assessment guided by the presenting condition | NA | Synchronous | VC and Telephone | Either eHAB or the Queensland Health Telehealth Portal |
| Salisbury et al., 2013^110^ | United Kingdom | Pragmatic RCT | n=1506 Age: median 48.27; IQR 36.72 to 61.01 | Senior physiotherapist trained in triaging over telephone | General musculoskeletal conditions presenting to primary care | Initial assessment and advice, and triage of patients | Education | Hybrid - asynchronous and synchronous | Telephone | NA |
| Sari et al., 2023^111^ | Australia | Pre-post intervention | n=30 Age: mean 71.5; SD 8.8 | Physiotherapist | Dementia | NA | Exercise | Synchronous | VC | Zoom |
| Sarmento et al., 2024^112^ | Canada | Pilot study | n=8 Age: mean 50; SD 9 | Physiotherapist | Post COVID syndrome | Subjective assessment, severity of COVID-19, self-reported physical activity level before COVID-19 and use of respiratory equipment during and after COVID-19 | Exercise Education | Synchronous | VC | Zoom |
| Seker et al., 2024^113^ | Turkey | RCT | n=40 Age: Mean 28.35; SD 3.56 | Physiotherapist | Healthy office workers | NA | Exercise | Synchronous | VC | NR |
| Sel et al., 2023^114^ | Turkey | RCT | n=23 Age: mean 4.63; SD 1.06 | Physiotherapist with seven years experience | Cerebral palsy | Gross Motor Function Measure, Canadian Occupational Performance Measure, Goal Attainment Scale, Paediatric Evaluation of Disability Inventory | Education Coaching | Hybrid - F2F (assessment) and synchronous (intervention) | VC | WhatsApp |
| Shih et al., 2022^115^ | United States of America | Cohort study | n=62 Age: mean 65.4; SD 9.2 | Physiotherapist | Idiopathic Parkinsons | NA | Exercise Coaching | Synchronous | VC | Zoom |
| Starzec-Proserpio and Malgorzata 2023^116^ | Canada | Case report | n=1 Age: 26 | Physiotherapist | Persistent pelvic girdle pain post partum | range of motion  Self-administered self-provocation tests self palpation, dynamic posture and movement assessment, gait analysis. | Exercise Education | Synchronous | VC | Zoom for Healthcare embedded within Embodia |
| Stavrakidou et al., 2023^117^ | Greece | RCT | n=15 Age: median 12; range 8 - 16 | Specialised paediatric physiotherapist | Juvenile idiopathic arthritis (polyarticular and oligoarticular) | NA | Exercise Education | Synchronous | VC | Skype |
| Sudini et al., 2022^118^ | India | Case study | n=1 Age: 27 | Pelvic floor physiotherapist | Dyspareunia - vaginismus associated with hypertonic pelvic floor muscles | Subjective assessment , Pelvic floor muscle strength testing | Exercise Coaching | Synchronous | VC | NR |
| Tarakci et al., 2021^119^ | Turkey | Randomised single blind study | n=15 Age: mean 39; SD 11 | Physiotherapist | Multiple sclerosis | NA | Exercise Coaching | Hybrid - synchronous and F2F | VC | NR |
| Tatemoto et al., 2022^120^ | Japan | Case series | n=2 Age: 72 & 49 | Physiotherapist with 12 years of experience | COVID, one with background of post cervical cord tumour removal, neurofibromatosis & deafness, other spoke different language | Vital signs and basic motor skill assessment | Exercise | Synchronous | VC | Zoom and Skype, with Teamviewer for remote control of device. |
| Timurtas et al., 2024^121^ | Turkey | RCT | n=30 Age: mean 46.1; SD 11.4 | Physiotherapist | Non-specific neck pain | NA | Exercise | Synchronous | VC | Zoom |
| Tore et al., 2023^122^ | NR | RCT | n=24 Age: mean 55.87; SD 7.24 | Physiotherapist | Mild/moderate knee osteoarthritis | 30sec chair stand test | Exercise Education | Synchronous | VC | Zoom |
| Truter et al., 2014^123^ | Australia | Validation study | n=26 Age: mean 43 | Rural physiotherapists | Current of recent low back pain | Standing posture assessment, ROM, straight leg raise test | NA | Synchronous | VC | eHAB TR system |
| Tsai et al., 2017^124^ | Australia | RCT | n=20 Age: mean 73; SD 8 | Hospital physiotherapist | Chronic obstructive pulmonary disease | NA | Exercise | Synchronous | VC | Vsee |
| Turcinovic et al., 2021^125^ | United States of America | Prospective quality improvement trial | n=32 Age: mean 57.3; SD 12.4 | Hospital physiotherapist | COVID | NA | Exercise | Hybrid - F2F and synchronous | VC | Avizia |
| van Egmond et al., 2020^126^ | Netherlands | Prospective feasibility study with matched historical comparison group | n=22 Age: mean 64.55; SD 6.72 | Physiotherapist | Post esophagectomy for oesophageal cancer, with postoperative complications | NA | Exercise Coaching | Hybrid - asynchronous and synchronous and F2F | Telephone and/or VC | Telephone and NR |
| Van Straaten et al., 2014^127^ | United States of America | Pre-post trial | n=16 Age: mean 41; range 25 - 64 | Physiotherapist | Manual wheelchair users with a spinal cord injury or post polio, and with shoulder pain | NA | Exercise Coaching | Hybrid - asynchronous and synchronous and F2F | VC | Skype |
| Wakasa et al., 2020^128^ | Japan | Pre-post trial | n=9 Age: mean 76.3; SD 3.3 | Physiotherapist | Older adults with no falls in last 12 months | NA | Exercise | Synchronous | VC | Live On |
| Weissman et al., 2023^129^ | United States of America | Case series | n=3 Age: 61, 36, 75 | Physiotherapist | Musculoskeletal conditions causing pain and participation restrictions (neck and shoulder pain with carpal tunnel syndrome, patellofemoral pain, femoral acetabular joint pain and L4-5 facet referred pain) | Subjective examination, ROM, patient administered special tests Tinels test, muscle length testing | Exercise Education | Hybrid - synchronous and F2F (2 cases); Synchronous only (1 case) | Telephone (assessment) and VC (assessment and intervention) | NR |
| Wood et al., 2016^130^ | United Kingdom | Pre-post trial | n=58 Age: mean 68.7; SD 7.8 | Physiotherapist | Knee pain with reduced ROM, weakness, or reduced balance | NA | Coaching | Hybrid - synchronous and F2F | Telephone | NA |
| Yavas et al., 2023^131^ | Turkey | 3-armed RCT | n=15 Age: Mean 40.1; SD 9.88 | Physiotherapist | Multiple sclerosis and urgency urinary incontinence | NA | Exercises | Hybrid - Synchronous and F2F | VC | Skype |
| Zanaboni et al., 2023^132^ | Norway, Australia and Denmark | RCT | n=40 Age: mean 64.9; SD 7.1 | Physiotherapist specialised in pulmonary rehabilitation | Chronic obstructive pulmonary disease | NA | Exercise | Hybrid - F2F and synchronous and asynchronous | VC | Acano |
| *Zanaboni et al., 2013 & 2017^133, 134^ | Norway | Feasibility study | n=10 Age: mean 55.2; SD 6.1 | Physiotherapist | Chronic obstructive pulmonary disease | NA | Exercise Education | Synchronous | VC | Lifesize |

RCT= Randomised Controlled Trial; NA= Not applicable; NR= Not Reported; COVID= Coronavirus; VC= videoconferencing; F2F= Face to Face; ROM= Range of Motion; MDT= Multi- disciplinary team; BBS= Berg Balance Scale; Ex= exercise; GMFM-88= Gross Motor Function Measure 88 question version; SD= standard deviation; IQR= inter quartile range; *studies combined to avoid overrepresentation of the same cohort

**References.**

1. Adhikari SP, Shrestha P, Dev R. Feasibility and Effectiveness of Telephone-Based Telephysiotherapy for Treatment of Pain in Low-Resource Setting: A Retrospective Pre-Post Design. *Pain research & management*. 2020;2020:2741278. doi:<https://dx.doi.org/10.1155/2020/2741278>

2. Aktan R, Yilmaz H, Demir I, Ozalevli S. Agreement between tele-assessment and face-to-face assessment of 30-s sit-to-stand test in patients with type 2 diabetes mellitus. *Irish journal of medical science*. 2023;192(5):2173-2178. doi:<https://dx.doi.org/10.1007/s11845-022-03238-w>

3. Aloraini SM. COVID-19 pandemic and physiotherapy practice: the efficacy of a telephone-based therapeutic program for people with knee osteoarthritis. *Physiotherapy theory and practice*. 2023;39(3):479-489. doi:<https://dx.doi.org/10.1080/09593985.2021.2023925>

4. Alsobayel H, Alodaibi F, Albarrati A, Alsalamah N, Alhawas F, Alhowimel A. Does Telerehabilitation Help in Reducing Disability among People with Musculoskeletal Conditions? A Preliminary Study. *International journal of environmental research and public health*. 2021;19(1)doi:<https://dx.doi.org/10.3390/ijerph19010072>

5. Anghelescu A. Telerehabilitation: A Practical Remote Alternative for Coaching and Monitoring Physical Kinetic Therapy in Patients with Mild and Moderate Disabling Parkinson's Disease during the COVID-19 Pandemic. *Parkinson's Disease*. 2022;2022:4370712. doi:<https://dx.doi.org/10.1155/2022/4370712>

6. Baehr LA, Hiremath SV, Bruneau M, et al. Effect of Tele-exercise to Promote Empowered Movement for Individuals With Spinal Cord Injury (TEEMS) Program on Physical Activity Determinants and Behavior: A Mixed Methods Assessment. *Archives of Physical Medicine & Rehabilitation*. 2024;105(1):101-111. doi:10.1016/j.apmr.2023.08.019

7. Baehr LA, Kaimal G, Bruneau M, Finley M. Development and Feasibility of a Group Tele-Exercise Program for Individuals With Spinal Cord Injury. *Journal of Neurologic Physical Therapy*. 2023;47(4):200-207. doi:10.1097/NPT.0000000000000449

8. Batalik L, Konecny V, Dosbaba F, Vlazna D, Brat K. Cardiac Rehabilitation Based on the Walking Test and Telerehabilitation Improved Cardiorespiratory Fitness in People Diagnosed with Coronary Heart Disease during the COVID-19 Pandemic. *International journal of environmental research and public health*. 2021;18(5)doi:<https://dx.doi.org/10.3390/ijerph18052241>

9. Batsis JA, Petersen CL, Clark MM, et al. Feasibility and acceptability of a technology-based, rural weight management intervention in older adults with obesity. *BMC geriatrics*. 2021;21(1):44. doi:<https://dx.doi.org/10.1186/s12877-020-01978-x>

10. Bennell KL, Lawford BJ, Keating C, et al. Comparing Video-Based, Telehealth-Delivered Exercise and Weight Loss Programs With Online Education on Outcomes of Knee Osteoarthritis : A Randomized Trial. *Annals of internal medicine*. 2022;175(2):198-209. Comment in: J Physiother. 2023 Jul;69(3):190 PMID: 37230819 [<https://www.ncbi.nlm.nih.gov/pubmed/37230819>] Comment in: J Physiother. 2023 Jul;69(3):190 PMID: 37286386 [<https://www.ncbi.nlm.nih.gov/pubmed/37286386>]. doi:<https://dx.doi.org/10.7326/M21-2388>

11. Benz C, Middleton A, Elliott A, Harvey A. Physiotherapy via telehealth for acute respiratory exacerbations in paediatric cystic fibrosis. *Journal of telemedicine and telecare*. 2023;29(7):552-560. doi:<https://dx.doi.org/10.1177/1357633X21998205>

12. Beresford L, Norwood T. Can Physical Therapy Deliver Clinically Meaningful Improvements in Pain and Function Through a Mobile App? An Observational Retrospective Study. *Archives of Rehabilitation Research and Clinical Translation*. 2022;4(2):100186. doi:<https://dx.doi.org/10.1016/j.arrct.2022.100186>

13. Bernocchi P, Vanoglio F, Baratti D, et al. Home-based telesurveillance and rehabilitation after stroke: a real-life study. *Topics in stroke rehabilitation*. 2016;23(2):106-15. doi:<https://dx.doi.org/10.1080/10749357.2015.1120453>

14. Bernocchi P, Vitacca M, La Rovere MT, et al. Home-based telerehabilitation in older patients with chronic obstructive pulmonary disease and heart failure: a randomised controlled trial. *Age and ageing*. 2018;47(1):82-88. doi:<https://dx.doi.org/10.1093/ageing/afx146>

15. Bickton FM, Chisati E, Rylance J, Morton B. An Improvised Pulmonary Telerehabilitation Program for Postacute COVID-19 Patients Would Be Feasible and Acceptable in a Low-Resource Setting. *American Journal of Physical Medicine & Rehabilitation*. 2021;100(3):209-212. doi:10.1097/PHM.0000000000001666

16. Blioumpa C, Karanasiou E, Antoniou V, et al. Efficacy of supervised home-based, real time, videoconferencing telerehabilitation in patients with type 2 diabetes: a single-blind randomized controlled trial. *European journal of physical and rehabilitation medicine*. 2023;59(5):628-639. doi:<https://dx.doi.org/10.23736/S1973-9087.23.07855-3>

17. Bowman A, Denehy L, Benjemaa A, et al. Feasibility and safety of the 30-second sit-to-stand test delivered via telehealth: An observational study. *PM & R : the journal of injury, function, and rehabilitation*. 2023;15(1):31-40. doi:<https://dx.doi.org/10.1002/pmrj.12783>

18. Brennan L, Sadeghi F, O'Neill L, et al. Telehealth Delivery of a Multi-Disciplinary Rehabilitation Programme for Upper Gastro-Intestinal Cancer: ReStOre@Home Feasibility Study. *Cancers*. 2022;14(11):2707-2707. doi:10.3390/cancers14112707

19. Brennen R, Soh S-E, Denehy L, et al. Pelvic floor muscle training delivered via telehealth to treat urinary and/or faecal incontinence after gynaecological cancer surgery: a single cohort feasibility study. *Supportive care in cancer : official journal of the Multinational Association of Supportive Care in Cancer*. 2023;31(10):589. doi:<https://dx.doi.org/10.1007/s00520-023-08050-5>

20. Brouwers RWM, van der Poort EKJ, Kemps HMC, van den Akker-van Marle ME, Kraal JJ. Cost-effectiveness of Cardiac Telerehabilitation With Relapse Prevention for the Treatment of Patients With Coronary Artery Disease in the Netherlands. *JAMA network open*. 2021;4(12):e2136652. Erratum in: JAMA Netw Open. 2022 Jan 4;5(1):e2147432 PMID: 35029668 [<https://www.ncbi.nlm.nih.gov/pubmed/35029668>]. doi:<https://dx.doi.org/10.1001/jamanetworkopen.2021.36652>

21. Bulguroglu HI, Bulguroglu M. The effects of online pilates and face-to-face pilates in healthy individuals during the COVID-19 pandemic: a randomized controlled study. *BMC Sports Science, Medicine and Rehabilitation*. 2023;15(1):12. doi:<https://dx.doi.org/10.1186/s13102-023-00625-3>

22. Campbell KR, Wilhelm JL, Pettigrew NC, Scanlan KT, Chesnutt JC, King LA. Implementation and Adoption of Telerehabilitation for Treating Mild Traumatic Brain Injury. *Journal of neurologic physical therapy : JNPT*. 2022;46(4):E1-E10. doi:<https://dx.doi.org/10.1097/NPT.0000000000000409>

23. Cheville AL, Moynihan T, Herrin J, Loprinzi C, Kroenke K. Effect of Collaborative Telerehabilitation on Functional Impairment and Pain Among Patients With Advanced-Stage Cancer: A Randomized Clinical Trial. *JAMA oncology*. 2019;5(5):644-652. doi:<https://dx.doi.org/10.1001/jamaoncol.2019.0011>

24. Colombage UN, Soh S-E, Lin K-Y, Kruger J, Frawley HC. The feasibility of pelvic floor training to treat urinary incontinence in women with breast cancer: a telehealth intervention trial. *Breast cancer (Tokyo, Japan)*. 2023;30(1):121-130. doi:<https://dx.doi.org/10.1007/s12282-022-01405-6>

25. Conlan L, Thompson J, Fary R. An exploration of the efficacy of telehealth in the assessment and management of stress urinary incontinence among women in rural locations. *Australian & New Zealand Continence Journal*. Spring2016 2016;22(3):58-64.

26. Cooley Hidecker MJ, Landers MR, Piccorelli A, Bush E, Singh R. Coordinated speech therapy, physiotherapy, and pharmaceutical care telehealth for people with Parkinson disease in rural communities: an exploratory, 8-week cohort study for feasibility, safety, and signal of efficacy. *Rural and remote health*. 2022;22(1):6679. doi:<https://dx.doi.org/10.22605/RRH6679>

27. Coronado RA, Devin CJ, Pennings JS, et al. *Safety and feasibility of an early telephone-supported home exercise program after anterior cervical discectomy and fusion: a case series*. Vol. 37. 2021:1096-1108. 0959-3985. <https://search.ebscohost.com/login.aspx?direct=true&AuthType=shib&db=ccm&AN=152450095&site=ehost-live&scope=site&custid=s8849760>

28. Coronado RA, Sterling EK, Fenster DE, et al. Cognitive-behavioral-based physical therapy to enhance return to sport after anterior cruciate ligament reconstruction: An open pilot study. *Physical therapy in sport : official journal of the Association of Chartered Physiotherapists in Sports Medicine*. 2020;42:82-90. doi:<https://dx.doi.org/10.1016/j.ptsp.2020.01.004>

29. Cottrell MA, O'Leary SP, Swete-Kelly P, et al. Agreement between telehealth and in-person assessment of patients with chronic musculoskeletal conditions presenting to an advanced-practice physiotherapy screening clinic. *Musculoskeletal science & practice*. 2018;38:99-105. doi:<https://dx.doi.org/10.1016/j.msksp.2018.09.014>

30. Cronin E, Hickey P, Lynch P, Monaghan K. Feasibility, safety and outcomes of an online, remotely supervised neuropilates class in a post stroke patient: A case study. *Physiotherapy Practice & Research*. 2022;43(1):113-124. doi:10.3233/PPR-220641

31. de la Plaza San Frutos M, Abuin Porras V, Blanco Morales M, Arrabe MG, Estrada Barranco C, Rubio Alonso M. Telemedicine in pulmonary rehabilitation - benefits of a telerehabilitation program in post-COVID-19 patients: a controlled quasi-experimental study. *Therapeutic advances in respiratory disease*. 2023;17:17534666231167354. doi:<https://dx.doi.org/10.1177/17534666231167354>

32. Duruturk N, Ozkoslu MA. Effect of tele-rehabilitation on glucose control, exercise capacity, physical fitness, muscle strength and psychosocial status in patients with type 2 diabetes: A double blind randomized controlled trial. *Primary care diabetes*. 2019;13(6):542-548. doi:<https://dx.doi.org/10.1016/j.pcd.2019.03.007>

33. Ekmekyapar Firat Y, Turgay T, Sogan SS, Gunel Karadeniz P. Effects of LSVT-BIG via telerehabilitation on non-motor and motor symptoms and quality of life in Parkinson's disease. *Acta neurologica Belgica*. 2023;123(1):207-214. doi:<https://dx.doi.org/10.1007/s13760-022-02104-x>

34. Eldemir S, Guclu-Gunduz A, Eldemir K, Saygili F, Yilmaz R, Akbostanci MC. The effect of task-oriented circuit training-based telerehabilitation on upper extremity motor functions in patients with Parkinson's disease: A randomized controlled trial. *Parkinsonism & related disorders*. 2023;109:105334. doi:<https://dx.doi.org/10.1016/j.parkreldis.2023.105334>

35. Eyuboglu F, Inal-Ince D, Karamancioglu B, et al. Effect of tele-yoga on aerobic capacity, respiratory muscle strength, and cognitive performance in patients with obstructive sleep apnea syndrome. *Heart & Lung*. 2023;62:157-167. doi:10.1016/j.hrtlng.2023.07.005

36. Filakova K, Janikova A, Felsoci M, et al. Home-based cardio-oncology rehabilitation using a telerehabilitation platform in hematological cancer survivors: a feasibility study. *BMC Sports Science, Medicine and Rehabilitation*. 2023;15(1):38. doi:<https://dx.doi.org/10.1186/s13102-023-00650-2>

37. Flynn A, Preston E, Dennis S, Canning CG, Allen NE. Home-based exercise monitored with telehealth is feasible and acceptable compared to centre-based exercise in Parkinson's disease: A randomised pilot study. *Clinical rehabilitation*. 2021;35(5):728-739. doi:<https://dx.doi.org/10.1177/0269215520976265>

38. Gagnon M, Marino Merlo G, Yap R, et al. Using Telerehabilitation to Deliver a Home Exercise Program to Youth With Arthrogryposis: Single Cohort Pilot Study. *Journal of medical Internet research*. 2021;23(7):e27064. doi:<https://dx.doi.org/10.2196/27064>

39. Galiano-Castillo N, Ariza-Garcia A, Cantarero-Villanueva I, Fernandez-Lao C, Sanchez-Salado C, Arroyo-Morales M. Agreement between telerehabilitation involving caregivers and face-to-face clinical assessment of lymphedema in breast cancer survivors. *Supportive care in cancer : official journal of the Multinational Association of Supportive Care in Cancer*. 2014;22(1):253-8. doi:<https://dx.doi.org/10.1007/s00520-013-1971-8>

40. Gavazzi F, Adang L, Waldman A, et al. Reliability of the Telemedicine Application of the Gross Motor Function Measure-88 in Patients With Leukodystrophy. *Pediatric neurology*. 2021;125:34-39. doi:<https://dx.doi.org/10.1016/j.pediatrneurol.2021.09.012>

41. Gerguz C, Aras Bayram G. Effects of Yoga Training Applied with Telerehabilitation on Core Stabilization and Physical Fitness in Junior Tennis Players: A Randomized Controlled Trial. *Auswirkungen von Yogatraining in Verbindung mit Telerehabilitation auf die Rumpfstabilisierung und die korperliche Fitness bei jungen Tennisspielern: Eine randomisierte kontrollierte Studie*. 2023;30(5):431-439. doi:<https://dx.doi.org/10.1159/000533848>

42. Gillespie D, MacLellan C, Ferguson-Pell M, Taeger A, Manns PJ. Balancing Access with Technology: Comparing In-Person and Telerehabilitation Berg Balance Scale Scores among Stroke Survivors. *Physiotherapy Canada*. 2021;73(3):276-285. doi:10.3138/ptc-2019-0095

43. Goode AP, Stark Taylor S, Hastings SN, Stanwyck C, Coffman CJ, Allen KD. Effects of a Home-Based Telephone-Supported Physical Activity Program for Older Adult Veterans With Chronic Low Back Pain. *Physical therapy*. 2018;98(5):369-380. doi:10.1093/ptj/pzy026

44. Gutierrez RO, Galan Del Rio F, Cano de la Cuerda R, Alguacil Diego IM, Gonzalez RA, Page JCM. A telerehabilitation program by virtual reality-video games improves balance and postural control in multiple sclerosis patients. *NeuroRehabilitation*. 2013;33(4):545-54. doi:<https://dx.doi.org/10.3233/NRE-130995>

45. Hamidfar R, Murris-Espin M, Mahot M, et al. Feasibility of home initiation of an airway clearance device (SIMEOX) by telecare in people with non-cystic fibrosis bronchiectasis: a pilot study. *BMJ open respiratory research*. 2023;10(1)doi:<https://dx.doi.org/10.1136/bmjresp-2023-001722>

46. Hinman RS, Nelligan RK, Bennell KL, Delany C. "Sounds a Bit Crazy, But It Was Almost More Personal:" A Qualitative Study of Patient and Clinician Experiences of Physical Therapist-Prescribed Exercise For Knee Osteoarthritis Via Skype. *Arthritis care & research*. 2017;69(12):1834-1844. doi:<https://dx.doi.org/10.1002/acr.23218>

47. Hinman RS, Campbell PK, Lawford BJ, et al. Does telephone-delivered exercise advice and support by physiotherapists improve pain and/or function in people with knee osteoarthritis? Telecare randomised controlled trial. *British journal of sports medicine*. 2020;54(13):790-797. doi:<https://dx.doi.org/10.1136/bjsports-2019-101183>

48. Holland AE, Hill CJ, Rochford P, Fiore J, Berlowitz DJ, McDonald CF. Telerehabilitation for people with chronic obstructive pulmonary disease: feasibility of a simple, real time model of supervised exercise training. *Journal of Telemedicine & Telecare*. 2013;19(4):222-226. doi:10.1177/1357633X13487100

49. Hong M, Topete M, Yang M, Bailey JF. Effects of a Digital Musculoskeletal Acute Care Program on Chronic Pain Prevention: An Observational Study with Nonparticipant Comparison Group. *Journal of Pain Research*. 2022;15:3605-3613. doi:<https://dx.doi.org/10.2147/JPR.S385134>

50. Horton BS, Marland JD, West HS, Wylie JD. Transition to Telehealth Physical Therapy After Hip Arthroscopy for Femoroacetabular Impingement. *Orthopaedic Journal of Sports Medicine*. 2021;9(4):1-6. doi:10.1177/2325967121997469

51. HuzmelÍ ED, DemÍR YP, Aksay U, Korkmaz NC, BalcÍ NC, Kus G. Effects of Telerehabilitation on Flexibility, Abdominal Muscle Strength, Balance Function and Quality of Life in Healthy Sedentary Housewives: A Pilot Study. *Romanian Journal of Physical Therapy / Revista Romana de Kinetoterapie*. 2022;28(49):15-27.

52. Hwang R, Bruning J, Morris NR, Mandrusiak A, Russell T. Home-based telerehabilitation is not inferior to a centre-based program in patients with chronic heart failure: a randomised trial. *Journal of physiotherapy*. 2017;63(2):101-107. doi:<https://dx.doi.org/10.1016/j.jphys.2017.02.017>

53. Janela D, Areias AC, Molinos M, et al. Digital Care Program for Urinary Incontinence in Females: A Large-Scale, Prospective, Cohort Study. *Healthcare (2227-9032)*. 2024;12(2):141. doi:10.3390/healthcare12020141

54. Jasper A, Karim R, Uy SJM, et al. Validity and Reliability of Virtually Obtained Functional Outcomes in Older Adults: A Pilot Study. *Topics in Geriatric Rehabilitation*. 2023;39(4):235-239. doi:10.1097/TGR.0000000000000407

55. Kamali S, Ozengin N, Topcuoglu MA. The effect of e-pelvic floor muscle training on symptoms in women with stress urinary incontinence: a randomized controlled trial. *Women & health*. 2023;63(6):473-483. doi:<https://dx.doi.org/10.1080/03630242.2023.2223729>

56. Kenis-Coskun O, Imamoglu S, Karamancioglu B, Kurt K, Ozturk G, Karadag-Saygi E. Comparison of telerehabilitation versus home-based video exercise in patients with Duchenne muscular dystrophy: a single-blind randomized study. *Acta neurologica Belgica*. 2022;122(5):1269-1280. doi:<https://dx.doi.org/10.1007/s13760-022-01975-4>

57. Kepenek-Varol B, Zeren M, Dincer R, Erkaya S. Breathing and Relaxation Exercises Help Improving Fear of COVID-19, Anxiety, and Sleep Quality: A Randomized Controlled Trial. *Journal of integrative and complementary medicine*. 2022;28(7):579-586. doi:<https://dx.doi.org/10.1089/jicm.2021.0381>

58. Kinder J, Davenport T, Chong W. Lee A. Telerehabilitation for Treating Pelvic Floor Dysfunction: A Case Series of 3 Patients' Experiences. *Journal of Women's Health Physical Therapy*. 2019;43(1):44-50. doi:10.1097/JWH.0000000000000120

59. Kraal JJ, Peek N, Van den Akker-Van Marle ME, Kemps HM. Effects of home-based training with telemonitoring guidance in low to moderate risk patients entering cardiac rehabilitation: short-term results of the FIT@Home study. *European journal of preventive cardiology*. 2014;21(2 Suppl):26-31. doi:<https://dx.doi.org/10.1177/2047487314552606>

60. Kratz AL, Atalla M, Whibley D, Myles A, Thurston T, Fritz NE. Calling Out MS Fatigue: Feasibility and Preliminary Effects of a Pilot Randomized Telephone-Delivered Exercise Intervention for Multiple Sclerosis Fatigue. *Journal of neurologic physical therapy : JNPT*. 2020;44(1):23-31. Comment in: J Neurol Phys Ther. 2020 Jan;44(1):32-33 PMID: 31834218 [<https://www.ncbi.nlm.nih.gov/pubmed/31834218>]. doi:<https://dx.doi.org/10.1097/NPT.0000000000000296>

61. Lafaro KJ, Raz DJ, Kim JY, et al. Pilot study of a telehealth perioperative physical activity intervention for older adults with cancer and their caregivers. *Supportive care in cancer : official journal of the Multinational Association of Supportive Care in Cancer*. 2020;28(8):3867-3876. doi:<https://dx.doi.org/10.1007/s00520-019-05230-0>

62. Lavoie V, Bouchard M, Turcotte S, Tousignant M. Telerehabilitation for Individuals with Parkinson's Disease and a History of Falls: A Pilot Study. *Physiotherapy Canada*. Fall2021 2021;73(4):343-350. doi:10.3138/ptc-2019-0108

63. Lawford BJ, Delany C, Bennell KL, Hinman RS. "I was really sceptical...But it worked really well": a qualitative study of patient perceptions of telephone-delivered exercise therapy by physiotherapists for people with knee osteoarthritis. *Osteoarthritis and cartilage*. 2018;26(6):741-750. doi:<https://dx.doi.org/10.1016/j.joca.2018.02.909>

64. Lawford BJ, Dobson F, Bennell KL, et al. Clinician-administered performance-based tests via telehealth in people with chronic lower limb musculoskeletal disorders: Test–retest reliability and agreement with in-person assessment. *Journal of telemedicine and telecare*. 2022:1357633X221137387-1357633X221137387. doi:10.1177/1357633X221137387

65. Lawford BJ, Bennell KL, Campbell PK, Kasza J, Hinman RS. Therapeutic Alliance Between Physical Therapists and Patients With Knee Osteoarthritis Consulting Via Telephone: A Longitudinal Study. *Arthritis care & research*. 2020;72(5):652-660. doi:<https://dx.doi.org/10.1002/acr.23890>

66. Lawford BJ, Hinman RS, McManus F, et al. How Does Exercise, With and Without Diet, Improve Pain and Function in Knee Osteoarthritis? A Secondary Analysis of a Randomized Controlled Trial Exploring Potential Mediators of Effects. *Arthritis care & research*. 2023;75(11):2316-2327. doi:<https://dx.doi.org/10.1002/acr.25140>

67. Le Berre M, Filiatrault J, Reichetzer B, Dumoulin C. Group-Based Pelvic Floor Telerehabilitation to Treat Urinary Incontinence in Older Women: A Feasibility Study. *International journal of environmental research and public health*. 2023;20(10)doi:<https://dx.doi.org/10.3390/ijerph20105791>

68. Lee JH, Shin KH, Lee GB, Son S, Jang K-M. Comparison of Functional Outcomes between Supervised Rehabilitation and Telerehabilitation in Female Patients with Patellofemoral Pain Syndrome during the COVID-19 Pandemic. *International journal of environmental research and public health*. 2023;20(3)doi:<https://dx.doi.org/10.3390/ijerph20032233>

69. Lee Y, Jung KB. Effect of Physiotherapy to Correct Rounded Shoulder Posture in 30 Patients During the COVID-19 Pandemic in South Korea Using a Telerehabilitation Exercise Program to Improve Posture, Physical Function, and Reduced Pain, with Evaluation of Patient Satisfaction. *Medical science monitor : international medical journal of experimental and clinical research*. 2022;28:e938926. doi:<https://dx.doi.org/10.12659/MSM.938926>

70. Lotan M, Downs J, Elefant C. A Pilot Study Delivering Physiotherapy Support for Rett Syndrome Using a Telehealth Framework Suitable for COVID-19 Lockdown. *Developmental neurorehabilitation*. 2021;24(6):429-434. doi:<https://dx.doi.org/10.1080/17518423.2021.1914762>

71. Lundgren KM, Langlo KAR, Salvesen O, et al. Feasibility of telerehabilitation for heart failure patients inaccessible for outpatient rehabilitation. *ESC heart failure*. 2023;10(4):2406-2417. doi:<https://dx.doi.org/10.1002/ehf2.14405>

72. Mani S, Sharma S, Singh DK. Concurrent validity and reliability of telerehabilitation-based physiotherapy assessment of cervical spine in adults with non-specific neck pain. *Journal of telemedicine and telecare*. 2021;27(2):88-97. doi:<https://dx.doi.org/10.1177/1357633X19861802>

73. Mantelatto Andrade R, Gomes Santana B, Verttu Schmidt A, et al. Effect of traditional rehabilitation programme versus telerehabilitation in adolescents with idiopathic scoliosis during the COVID-19 pandemic: a cohort study. *Journal of rehabilitation medicine*. 2024;56:jrm5343. doi:<https://dx.doi.org/10.2340/jrm.v56.5343>

74. Manzak Dursun AS, Ozyilmaz S, Ucgun H, Elmadag NM. The effect of Pilates-based exercise applied with hybrid telerehabilitation method in children with adolescent idiopathic scoliosis: A randomized clinical trial. *European journal of pediatrics*. 2024;183(2):759-767. doi:<https://dx.doi.org/10.1007/s00431-023-05340-2>

75. Martin I, Braem F, Baudet L, et al. Follow-up of functional exercise capacity in patients with COVID-19: It is improved by telerehabilitation. *Respiratory medicine*. 2021;183:106438. doi:<https://dx.doi.org/10.1016/j.rmed.2021.106438>

76. Master H, Coronado RA, Whitaker S, et al. Combining Wearable Technology and Telehealth Counseling for Rehabilitation After Lumbar Spine Surgery: Feasibility and Acceptability of a Physical Activity Intervention. *Physical therapy*. 2024;104(2)doi:<https://dx.doi.org/10.1093/ptj/pzad096>

77. Mehta SP, Kendall KM, Reasor CM. Virtual assessments of knee and wrist joint range motion have comparable reliability with face-to-face assessments. *Musculoskeletal care*. 2021;19(2):208-216. doi:<https://dx.doi.org/10.1002/msc.1525>

78. Mesa-Castrillon CI, Simic M, Ferreira ML, et al. Effectiveness of an eHealth-Delivered Program to Empower People With Musculoskeletal Pain in Rural Australia: A Randomized Controlled Trial. *Arthritis care & research*. 2024;76(4):570-581. doi:<https://dx.doi.org/10.1002/acr.25272>

79. Middleton A, Simpson KN, Bettger JP, Bowden MG. COVID-19 Pandemic and Beyond: Considerations and Costs of Telehealth Exercise Programs for Older Adults With Functional Impairments Living at Home-Lessons Learned From a Pilot Case Study. *Physical therapy*. 2020;100(8):1278-1288. doi:<https://dx.doi.org/10.1093/ptj/pzaa089>

80. Mobbs C, Spittle A, Johnston L. PreEMPT (Preterm infant Early intervention for Movement and Participation Trial): Feasibility outcomes of a randomised controlled trial. *Early human development*. 2022;166:105551. doi:<https://dx.doi.org/10.1016/j.earlhumdev.2022.105551>

81. Nambi G, Alghadier M, Vellaiyan A, et al. Role of Tele-Physical Therapy Training on Glycemic Control, Pulmonary Function, Physical Fitness, and Health-Related Quality of Life in Patients with Type 2 Diabetes Mellitus (T2DM) Following COVID-19 Infection—A Randomized Controlled Trial. *Healthcare (2227-9032)*. 2023;11(12):1791. doi:10.3390/healthcare11121791

82. Nelson M, Bourke M, Crossley K, Russell T. Telerehabilitation is non-inferior to usual care following total hip replacement — a randomized controlled non-inferiority trial. *Physiotherapy*. 2020;107:19-27. doi:10.1016/j.physio.2019.06.006

83. Nicola K, Waugh J, Charles E, Russell T. The feasibility and concurrent validity of performing the Movement Assessment Battery for Children - 2nd Edition via telerehabilitation technology. *Research in developmental disabilities*. 2018;77:40-48. doi:<https://dx.doi.org/10.1016/j.ridd.2018.04.001>

84. Nunez-Cortes R, Cruz-Montecinos C, Torreblanca-Vargas S, et al. Effectiveness of adding pain neuroscience education to telerehabilitation in patients with carpal tunnel syndrome: A randomized controlled trial. *Musculoskeletal science & practice*. 2023;67:102835. doi:<https://dx.doi.org/10.1016/j.msksp.2023.102835>

85. O'Neil J, Egan M, Marshall S, Bilodeau M, Pelletier L, Sveistrup H. The Impact of Two Telerehabilitation Supervision Schedules on Physical Activity, Mobility, and Balance Among People with Moderate to Severe Traumatic Brain Injury: A Mixed-Method Single-Subject Design. *Physiotherapy Canada*. 2023;75(2):118-131. doi:10.3138/ptc-2021-0040

86. Okpara C, Ioannidis G, Thabane L, et al. The Geras virtual frailty rehabilitation program to build resilience in older adults with frailty during COVID-19: a randomized feasibility trial. *Pilot and Feasibility Studies*. 2023;9(1):124. doi:<https://dx.doi.org/10.1186/s40814-023-01346-7>

87. Okudan B, Celik D, Koban O. The effectiveness of telerehabilitation-based exercise combined with pain neuroscience education for patients with facet joint arthrosis: A randomized controlled study. *European journal of pain (London, England)*. 2024;28(3):382-395. doi:<https://dx.doi.org/10.1002/ejp.2187>

88. Onan D, Ulger O, Martelletti P. Effects of spinal stabilization exercises delivered using telerehabilitation on outcomes in patients with chronic neck pain: a randomized controlled trial. *Expert review of neurotherapeutics*. 2023;23(3):269-280. doi:<https://dx.doi.org/10.1080/14737175.2023.2192870>

89. Ozlu A, Unver G, Tuna HI, Erdogan A. Effects of Interactive Telerehabilitation Practices in Office Workers with Chronic Nonspecific Neck Pain: Randomized Controlled Study. *Telemedicine journal and e-health : the official journal of the American Telemedicine Association*. 2024;30(2):438-447. doi:<https://dx.doi.org/10.1089/tmj.2023.0018>

90. Ozturk B, Duruturk N. Effect of telerehabilitation applied during COVID-19 isolation period on physical fitness and quality of life in overweight and obese individuals. *International journal of obesity (2005)*. 2022;46(1):95-99. doi:<https://dx.doi.org/10.1038/s41366-021-00965-5>

91. Palmcrantz S, Borg J, Sommerfeld D, et al. An interactive distance solution for stroke rehabilitation in the home setting - A feasibility study. *Informatics for health & social care*. 2017;42(3):303-320. doi:<https://dx.doi.org/10.1080/17538157.2016.1253015>

92. Paolucci T, de Sire A, Ferrillo M, et al. Telerehabilitation proposal of mind-body technique for physical and psychological outcomes in patients with fibromyalgia. *Frontiers in Physiology*. 2022;13:917956. doi:<https://dx.doi.org/10.3389/fphys.2022.917956>

93. Park S, Tang A, Barclay R, et al. Investigating the Telerehabilitation With Aims to Improve Lower Extremity Recovery Poststroke Program: A Feasibility Study. *Physical therapy*. 2024;104(3)doi:<https://dx.doi.org/10.1093/ptj/pzad165>

94. Pastana Ramos LF, Vilacorta-Pereira TDCS, Duarte JDS, Yamada ES, Santos-Lobato BL. Feasibility and effectiveness of a remote individual rehabilitation program for people with Parkinson's disease living in the Brazilian Amazon: a randomized clinical trial. *Frontiers in Neurology*. 2023;14:1244661. doi:<https://dx.doi.org/10.3389/fneur.2023.1244661>

95. Pehlivan E, Palalı İ, Atan S, Turan D, Çınarka H, Çetinkaya E. The effectiveness of POST-DISCHARGE telerehabilitation practices in COVID-19 patients: Tele-COVID study-randomized controlled trial. *Annals of Thoracic Medicine*. 2022;17(2):110-117. doi:10.4103/atm.atm_543_21

96. Peterson S. Telerehabilitation booster sessions and remote patient monitoring in the management of chronic low back pain: A case series. *Physiotherapy theory and practice*. 2018;34(5):393-402. doi:<https://dx.doi.org/10.1080/09593985.2017.1401190>

97. Peterson S. Digital physical therapy practice and payment during the COVID-19 pandemic: A case series. *Physiotherapy theory and practice*. 2023;39(2):469-478. doi:<https://dx.doi.org/10.1080/09593985.2021.2021572>

98. Petter Rodrigues M, Soares Peterson B, Mallmann S, Laureano Paiva L, Lopes Ramos JG. Digital physical therapy intervention to treat female urinary incontinence: a semiexperimental study. *Fisioterapia Brasil*. 2023;24(5):647-660. doi:10.33233/fb.v24i5.5448

99. Pinto C, Figueiredo C, Mabilia V, Cruz T, Jeffrey ER, Souza Pagnussat A. A Safe and Feasible Online Dance Intervention for Older Adults With and Without Parkinson's Disease. *Journal of Dance Medicine & Science*. 2023;27(4):253-267. doi:10.1177/1089313X231186201

100. Piraux E, Reychler G, Forget P, Yombi J-C, Caty G. Feasibility and Preliminary Effects of a Telerehabilitation Program for People Living With HIV: A Pilot Randomized Study. *The Journal of the Association of Nurses in AIDS Care : JANAC*. 2019;30(2):176-185. doi:<https://dx.doi.org/10.1097/JNC.0000000000000005>

101. Plaza A, Paratz J, Cottrell M. A six-week physical therapy exercise program delivered via home-based telerehabilitation is comparable to in-person programs for patients with burn injuries: A randomized, controlled, non-inferiority clinical pilot trial. *Burns : journal of the International Society for Burn Injuries*. 2023;49(1):55-67. doi:<https://dx.doi.org/10.1016/j.burns.2022.08.014>

102. Pleguezuelos E, Del Carmen A, Moreno E, Miravitlles M, Serra M, Garnacho-Castano MV. Effects of a telerehabilitation program and detraining on cardiorespiratory fitness in patients with post-COVID-19 sequelae: A randomized controlled trial. *Scandinavian journal of medicine & science in sports*. 2024;34(1):e14543. doi:<https://dx.doi.org/10.1111/sms.14543>

103. Post AA, Rio EK, Sluka KA, et al. Efficacy of Telehealth for Movement-Evoked Pain in People With Chronic Achilles Tendinopathy: A Noninferiority Analysis. *Physical therapy*. 2023;103(3)doi:<https://dx.doi.org/10.1093/ptj/pzac171>

104. Ramachandra P. Telerehabilitation for pelvic girdle dysfunction in pregnancy during COVID-19 pandemic crisis: A case report. *Physiotherapy theory and practice*. 2022;38(12):2250-2256. doi:<https://dx.doi.org/10.1080/09593985.2021.1898706>

105. Richardson BR, Truter P, Blumke R, Russell TG. Physiotherapy assessment and diagnosis of musculoskeletal disorders of the knee via telerehabilitation. *Journal of telemedicine and telecare*. 2017;23(1):88-95. doi:<https://dx.doi.org/10.1177/1357633X15627237>

106. Rodriguez-Blanco C, Bernal-Utrera C, Anarte-Lazo E, Gonzalez-Gerez JJ, Saavedra-Hernandez M. A 14-Day Therapeutic Exercise Telerehabilitation Protocol of Physiotherapy Is Effective in Non-Hospitalized Post-COVID-19 Conditions: A Randomized Controlled Trial. *Journal of Clinical Medicine*. 2023;12(3):776. doi:<https://dx.doi.org/10.3390/jcm12030776>

107. Rodriguez-Blanco C, Bernal-Utrera C, Anarte-Lazo E, et al. Breathing exercises versus strength exercises through telerehabilitation in coronavirus disease 2019 patients in the acute phase: A randomized controlled trial. *Clinical rehabilitation*. 2022;36(4):486-497. doi:<https://dx.doi.org/10.1177/02692155211061221>

108. Rosenbek Minet L, Hansen LW, Pedersen CD, et al. Early telemedicine training and counselling after hospitalization in patients with severe chronic obstructive pulmonary disease: a feasibility study. *BMC medical informatics and decision making*. 2015;15:3. doi:<https://dx.doi.org/10.1186/s12911-014-0124-4>

109. Russell S, Whitehart S, Mason J, Window P. Does the method of telehealth delivery affect the physiotherapy management of adults with bleeding disorders? A comparison of audioconferencing and videoconferencing. *Haemophilia : the official journal of the World Federation of Hemophilia*. 2023;29(6):1589-1596. doi:<https://dx.doi.org/10.1111/hae.14869>

110. Salisbury C, Foster N, Hopper C, et al. A pragmatic randomised controlled trial of the effectiveness and cost-effectiveness of 'PhysioDirect' telephone assessment and advice services for physiotherapy. *Health Technology Assessment*. 2013;17(32):1-157. doi:10.3310/hta17020

111. Sari YM, Burton E, Lee D-CA, Hill KD. A Telehealth Home-Based Exercise Program for Community-Dwelling Older People with Dementia in Indonesia: A Feasibility Study. *International journal of environmental research and public health*. 2023;20(4)doi:<https://dx.doi.org/10.3390/ijerph20043397>

112. Sarmento A, Adodo R, Hodges G, Webber SC, Sanchez-Ramirez DC. Virtual pulmonary rehabilitation approaches in patients with post COVID syndrome: a pilot study. *BMC Pulmonary Medicine*. 2024;24(1):139. doi:<https://dx.doi.org/10.1186/s12890-024-02965-3>

113. Seker AN, Arman N. Comparison of the Effects of Two Different Exercise Programs on Lower Limb Functions, Posture, and Physical Activity in Office Workers Working at Home and in Office Alternately. *American Journal of Physical Medicine & Rehabilitation*. 2024;103(2):134-142. doi:10.1097/PHM.0000000000002315

114. Sel SA, Gunel MK, Erdem S, Tuncdemir M. Effects of Telerehabilitation-Based Structured Home Program on Activity, Participation and Goal Achievement in Preschool Children with Cerebral Palsy: A Triple-Blinded Randomized Controlled Trial. *Children*. 2023;10(3):424. doi:<https://dx.doi.org/10.3390/children10030424>

115. Shih H-JS, Macpherson CE, King M, et al. Physical Activity Coaching via Telehealth for People With Parkinson Disease: A Cohort Study. *Journal of neurologic physical therapy : JNPT*. 2022;46(4):240-250. doi:<https://dx.doi.org/10.1097/NPT.0000000000000410>

116. Starzec-Proserpio M, Vandyken C. Telerehabilitation for persistent Pelvic Girdle Pain within a biopsychosocial framework - A case report. *Physiotherapy theory and practice*. 2023;39(10):2251-2261. doi:<https://dx.doi.org/10.1080/09593985.2022.2069618>

117. Stavrakidou M, Trachana M, Koutsonikoli A, Spanidou K, Hristara-Papadopoulou A. The Impact of a Physiotherapy Tele-Rehabilitation Program on the Quality of Care for Children with Juvenile Idiopathic Arthritis. *Mediterranean Journal of Rheumatology*. 2023;34(4):443-453. doi:<https://dx.doi.org/10.31138/mjr.310823.tio>

118. Sudini B, Binal D. A Tele Rehabilitation Approach for the Treatment of Dyspareunia: Case Report. *Indian Journal of Physiotherapy & Occupational Therapy*. 2022;16(2):44-48. doi:10.37506/ijpot.v16i2.18032

119. Tarakci E, Tarakci D, Hajebrahimi F, Budak M. Supervised exercises versus telerehabilitation. Benefits for persons with multiple sclerosis. *Acta neurologica Scandinavica*. 2021;144(3):303-311. doi:<https://dx.doi.org/10.1111/ane.13448>

120. Tatemoto T, Mukaino M, Kumazawa N, et al. Overcoming language barriers to provide telerehabilitation for COVID-19 patients: a two-case report. *Disability and rehabilitation Assistive technology*. 2022;17(3):275-282. doi:<https://dx.doi.org/10.1080/17483107.2021.2013962>

121. Timurtas E, Selcuk H, Ugur Canoz E, et al. Synchronous and asynchronous telerehabilitation methods produce similar benefits in individuals with non-specific neck pain. *Archives of orthopaedic and trauma surgery*. 2024;144(2):559-566. doi:<https://dx.doi.org/10.1007/s00402-023-05083-7>

122. Tore NG, Oskay D, Haznedaroglu S. The quality of physiotherapy and rehabilitation program and the effect of telerehabilitation on patients with knee osteoarthritis. *Clinical rheumatology*. 2023;42(3):903-915. doi:<https://dx.doi.org/10.1007/s10067-022-06417-3>

123. Truter P, Russell T, Fary R. The validity of physical therapy assessment of low back pain via telerehabilitation in a clinical setting. *Telemedicine journal and e-health : the official journal of the American Telemedicine Association*. 2014;20(2):161-7. doi:<https://dx.doi.org/10.1089/tmj.2013.0088>

124. Tsai LLY, McNamara RJ, Moddel C, Alison JA, McKenzie DK, McKeough ZJ. Home‐based telerehabilitation via real‐time videoconferencing improves endurance exercise capacity in patients with COPD: the randomized controlled TeleR Study. *Respirology*. 2017;22(4):699-707.

125. Turcinovic M, Singson R, Harrigan M, et al. Physical Therapy for Hospitalized Patients With COVID-19 in Isolation: Feasibility and Pilot Implementation of Telehealth for Delivering Individualized Therapy. *Archives of Rehabilitation Research and Clinical Translation*. 2021;3(2):100113. doi:<https://dx.doi.org/10.1016/j.arrct.2021.100113>

126. van Egmond MA, Engelbert RHH, Klinkenbijl JHG, van Berge Henegouwen MI, van der Schaaf M. Physiotherapy With Telerehabilitation in Patients With Complicated Postoperative Recovery After Esophageal Cancer Surgery: Feasibility Study. *Journal of medical Internet research*. 2020;22(6):e16056. doi:<https://dx.doi.org/10.2196/16056>

127. Van Straaten MG, Cloud BA, Morrow MM, Ludewig PM, Zhao KD. Effectiveness of home exercise on pain, function, and strength of manual wheelchair users with spinal cord injury: a high-dose shoulder program with telerehabilitation. *Archives of physical medicine and rehabilitation*. 2014;95(10):1810-1817.e2. doi:<https://dx.doi.org/10.1016/j.apmr.2014.05.004>

128. Wakasa M, Odashima T, Saito A, et al. Telerehabilitation with Tablet Computers Replaces Face-to-Face Rehabilitation. *Physical & Occupational Therapy in Geriatrics*. 2020;38(1):85-97. doi:10.1080/02703181.2019.1660446

129. Weissman S, Gladin A, Davenport TE. Using Clinical Decision Making to Identify the Appropriateness of Telehealth Physical Therapy: A Case Series. *Orthopaedic Physical Therapy Practice*. 2023;35(1):31-34.

130. Wood LRJ, Blagojevic-Bucknall M, Stynes S, et al. Impairment-targeted exercises for older adults with knee pain: A proof-of-principle study (TargET-Knee-Pain). *BMC Musculoskeletal Disorders*. 2016;17(1):47. doi:<https://dx.doi.org/10.1186/s12891-016-0899-9>

131. Yavas I, Kahraman T, Sagici O, et al. Feasibility of Telerehabilitation-Based Pelvic Floor Muscle Training for Urinary Incontinence in People With Multiple Sclerosis: A Randomized, Controlled, Assessor-Blinded Study. *Journal of neurologic physical therapy : JNPT*. 2023;47(4):217-226. doi:<https://dx.doi.org/10.1097/NPT.0000000000000448>

132. Zanaboni P, Dinesen B, Hoaas H, et al. Long-term Telerehabilitation or Unsupervised Training at Home for Patients with Chronic Obstructive Pulmonary Disease: A Randomized Controlled Trial. *American journal of respiratory and critical care medicine*. 2023;207(7):865-875. Comment in: Am J Respir Crit Care Med. 2023 Apr 1;207(7):804-805 PMID: 36656552 [<https://www.ncbi.nlm.nih.gov/pubmed/36656552>]. doi:<https://dx.doi.org/10.1164/rccm.202204-0643OC>

133. Zanaboni P, Lien LA, Hjalmarsen A, Wootton R. Long-term telerehabilitation of COPD patients in their homes: interim results from a pilot study in Northern Norway. *Journal of telemedicine and telecare*. 2013;19(7):425-9. doi:<https://dx.doi.org/10.1177/1357633X13506514>

134. Zanaboni P, Hoaas H, Aaroen Lien L, Hjalmarsen A, Wootton R. Long-term exercise maintenance in COPD via telerehabilitation: a two-year pilot study. *Journal of telemedicine and telecare*. 2017;23(1):74-82. doi:<https://dx.doi.org/10.1177/1357633X15625545>
