# Supplementary material for: Informing the Development of Telehealth Education in Physiotherapy Programs. Assessments and Interventions for Individuals Accessing Physiotherapy Care via Synchronous Telehealth. A Scoping Review
Source: Musculoskeletal Care. 2025 Jan 9;23(1):e70039. doi: 10.1002/msc.70039 (PMC11717065; doi:10.1002/msc.70039)
Supplement: Supplementary file 2 — Supporting Information S2 [file MSC-23-e70039-s003.docx]

Appendix B. Telehealth platforms used

| **Platform (n=87)** | **n (%)** |
| --- | --- |
| Acano | 1 (1) |
| Adobe Connect | 1 (1) |
| American Well Platform | 1 (1) |
| Avizia | 1 (1) |
| Bespoke closed secure system | 1 (1) |
| BlueJay engage | 1 (1) |
| Cisco WebEx | 1 (1) |
| Salso Health Solution | 1 (1) |
| DISKO-tool | 1 (1) |
| Doxy.me | 1 (1) |
| eHAB | 7 (8) |
| Google Meet | 3 (3) |
| Lifesize | 2 (2) |
| Live On | 1 (1) |
| Microsoft Teams | 2 (2) |
| Not reported | 17 |
| Omada Health | 1 (1) |
| Physitrack | 1 (1) |
| Polar Flow | 1 (1) |
| PreEMPT | 1 (1) |
| SIMEOX | 1 (1) |
| Skype | 9 (10) |
| TelePTsys | 1 (1) |
| TeleRiab | 1 (1) |
| Teraplus | 1 (1) |
| Vidyo | 1 (1) |
| Vsee | 2 (2) |
| Webex | 1 (1) |
| WhatsApp | 10 (11) |
| Wormhole Web Conference | 1 (1) |
| Zoom | 30 (34) |
