# Supplementary material for: Informing the Development of Telehealth Education in Physiotherapy Programs. Assessments and Interventions for Individuals Accessing Physiotherapy Care via Synchronous Telehealth. A Scoping Review
Source: Musculoskeletal Care. 2025 Jan 9;23(1):e70039. doi: 10.1002/msc.70039 (PMC11717065; doi:10.1002/msc.70039)
Supplement: Supplementary file 3 — Supporting Information S3 [file MSC-23-e70039-s005.docx]

Appendix C. Assessments delivered via synchronous telehealth to different patient populations

| **Assessment** | **Musculoskeletal** | **Neurological** | **Respiratory** | **Cardiac** | **Oncology** | **Metabolic** | **Pelvic health** | **Healthy** | **Other** |
| --- | --- | --- | --- | --- | --- | --- | --- | --- | --- |
| Subjective assessment | B, D | - | C | - | D | - | B, C | - | - |
| Range of motion | A, B, C, D | - | - | - | - | - | B | B | - |
| Postural examination | B | - | - | - | - | - | B | - | - |
| Muscular endurance tests | B, D | - | - | - | - | - | - | - | - |
| Muscle length test | B, D | - | - | - | - | - | B | - | - |
| Straight leg raise | C | - | - | - | - | - | B | - | - |
| Pelvic floor muscle strength test | - | - | - | - | - | - | B | - | - |
| Arm curl test | - | - | - | - | - | C | - | - | - |
| 2-minute step test | D | - | - | D | - | C | - | - | D |
| Chair sit and reach test | - | - | - | - | - | C | - | - | - |
| Timed up and go | D | - | - | D | - | - | - | - | D |
| Sit to stand (30 sec, 1min) | D | - | B, D | D | D | C | B | - | D |
| 5 times sit to stand | - | D | B | - | D | - | B | - | - |
| Berg Balance | - | D | - | D | - | D | - | - | - |
| 5 Meter walk test | - | D | - | D | - | D | - | - | - |
| 6 Minute walk test | - | - | C | - | - | - | - | - | - |
| Stair climb test | D | - | - | - | - | - | - | - | - |
| Calf raise test | C | - | - | - | - | - | - | - | - |
| Abdominal curl up | - | - | - | - | - | - | B, C | - | - |
| Timed single leg stance | D | - | - | - | - | - | - | - | - |
| Heart rate, respiratory rate, oxygen saturation | - | - | C | - | - | - | - | - | - |
| Self-administered provocation tests | - | - | - | - | - | - | B | - | - |
| Self-administered palpation | B, D | - | - | - | - | - | B | - | - |
| Self-administered sensation tests | B, D | - | - | - | - | - | - | - | - |
| Self-administered orthopaedic tests (manual muscle test, shoulder test) | B, C | - | - | - | - | - | - | - | - |
| Modified special tests | B | - | - | - | - | - | - | - | - |
| Circumferential arm measurements | - | - | - | - | C | - | - | - | - |
| Motor skill assessment | - | C, D | - | - | - | - | - | - | - |
| Gross motor function measure | - | A | - | - | - | - | - | - | - |
| Movement assessment battery for children 2^nd^ edition | - | - | - | - | - | - | - | A | - |

A= 0 -20 years; B= 21 – 40 years; C= 41 – 60 years; D= 60+ years
