# Supplementary material for: Informing the Development of Telehealth Education in Physiotherapy Programs. Assessments and Interventions for Individuals Accessing Physiotherapy Care via Synchronous Telehealth. A Scoping Review
Source: Musculoskeletal Care. 2025 Jan 9;23(1):e70039. doi: 10.1002/msc.70039 (PMC11717065; doi:10.1002/msc.70039)
Supplement: Supplementary file 4 — Supporting Information S4 [file MSC-23-e70039-s001.docx]

Appendix D. Interventions delivered via synchronous telehealth to different patient populations

| **Intervention** | **Musculoskeletal** | **Neurological** | **Respiratory** | **Cardiac** | **Oncology** | **Metabolic** | **Pelvic health** | **Healthy** | **Other** |
| --- | --- | --- | --- | --- | --- | --- | --- | --- | --- |
| Exercise | A, B, C, D | A, B, C, D | A, B, C, D | A, C, D | C, D | C, D | B, C | B, C | A, C, D |
| Education | B, C, D | A, C, D | C, D | C, D | B | - | B, C | - | - |
| Coaching | - | D | - | C | D | - | - | - | - |

A= 0 – 20 years-; B= 21 – 40 years; C= 41 – 60 years; D= 61+ years
