# Supplementary material for: Informing the Development of Telehealth Education in Physiotherapy Programs. Assessments and Interventions for Individuals Accessing Physiotherapy Care via Synchronous Telehealth. A Scoping Review
Source: Musculoskeletal Care. 2025 Jan 9;23(1):e70039. doi: 10.1002/msc.70039 (PMC11717065; doi:10.1002/msc.70039)
Supplement: Supplementary file 5 — Supporting Information S5 [file MSC-23-e70039-s004.docx]

Appendix E. Conditions assessed and/or managed by physiotherapist via synchronous telehealth across population/practice area.

| **Musculoskeletal** | **Respiratory** | **Oncology** | **Neurological** | **Cardiac** | **Metabolic** | **Pelvic health** | **Healthy** | **Other** |
| --- | --- | --- | --- | --- | --- | --- | --- | --- |
| Acute musculoskeletal conditions  Anterior cruciate ligament  Cervical discectomy  Carpal tunnel syndrome  Achillies tendinopathy  Chronic low back pain / general low back pain  Chronic neck pain / general neck pain  Femoroacetabular impingement  General musculoskeletal conditions  Juvenile idiopathic arthritis  Knee pain  Osteoarthritis  Patellofemoral pain syndrome  Post operative (hip)  Traumatic ankle sprain | Acute COVID-19  COVID-19  Chronic obstructive pulmonary disease  Cystic fibrosis  Bronchiectasis  Long COVID  Post COVID syndrome | Breast cancer  Endometrial, uterine, cervical, ovarian cancer (stage I-III)  Oesophageal cancer  Gastrointestinal and lung cancer  Haematological cancer  Lymphoedema | Basal ganglia infarct  Cerebral palsy  Dementia  Duchenne’s muscular dystrophy  Fibromyalgia  Idiopathic Parkinson’s disease  Leukodystrophy  Mild traumatic brain injury  Multiple sclerosis  Premature birth  Spinal cord injury  Stroke | Chronic heart failure  Coronary artery disease  General cardiac issues  Myocardial infarction  Unstable angina | Obesity  Overweight  Type 2 diabetes mellitus | Dyspareunia  Diastasis recti  Hypertonic pelvic floor muscles  Pelvic girdle dysfunction  Premature ejaculation  Stress urinary incontinence | Healthy adults  Healthy office workers  Healthy university workers  Typically developing children  Older adults with no falls in past 12 months  Young athletes | Inherited bleeding disorders  Burn injuries  Human Immunodeficiency virus  Vulnerable frail older adults |
